# Risk of aortic aneurysm or dissection following use of fluoroquinolones: a retrospective multinational network cohort study

**DOI:** 10.1016/j.eclinm.2025.103096

**Published:** 2025-02-01

**Authors:** Jack L. Janetzki, Jung Ho Kim, Evan Minty, Jung Ah Lee, Daniel R. Morales, Rohan Khera, Chungsoo Kim, Thamir M. Alshammari, Scott L. DuVall, Michael E. Matheny, Thomas Falconer, Seonji Kim, Thanh-Phuc Phan, Phung-Anh Nguyen, Min-Huei Hsu, Jason C. Hsu, Rae Woong Park, Kenneth K.C. Man, Sarah Seager, Mui Van Zandt, James P. Gilbert, Patrick B. Ryan, Martijn J. Schuemie, Marc A. Suchard, George Hripcsak, Nicole Pratt, Seng Chan You

**Affiliations:** a Clinical and Health Sciences, Quality Use of Medicines and Pharmacy Research Centre, University of South Australia, Adelaide, Australia; b Department of Internal Medicine, Yonsei University College of Medicine, Seoul, South Korea; c Department of Medicine, University of Calgary, Calgary, Canada; d Division of Population Health and Genomics, University of Dundee, Dundee, United Kingdom; e Section of Cardiovascular Medicine, Department of Internal Medicine, Yale University, New Haven, USA; f Department of Clinical Pharmacy, Pharmacy Practice Research Unit, Faculty of Pharmacy, Jazan University, Jazan, Saudi Arabia; g VA Informatics and Computing Infrastructure, United States Department of Veterans Affairs, Salt Lake City, USA; h Tennessee Valley Healthcare System, Veterans Affairs Medical Center, Nashville, USA; i Department of Biomedical Informatics, Columbia University, New York, USA; j Department of Biomedical Systems Informatics, Yonsei University College of Medicine, Seoul, South Korea; k International Ph.D. Program in Biotech and Healthcare Management, College of Management, Taipei Medical University, New Taipei City, Taiwan; l Clinical Data Center, Office of Data Science, Taipei Medical University, New Taipei City, Taiwan; m Graduate Institute of Data Science, College of Management, Taipei Medical University, Taipei City, Taiwan; n Department of Biomedical Informatics, Ajou University School of Medicine, Suwon, South Korea; o School of Pharmacy, University College London, London, United Kingdom; p IQVIA, Cambridge, MA, USA; q Janssen Research and Development, Titusville, USA; r Epidemiology, Johnson & Johnson, Titusville, USA; s Department of Biostatistics, University of California, Los Angeles, USA

**Keywords:** Fluoroquinolone, Observational study, Aortic dissection, Aortic aneurysm

## Abstract

**Background:**

Fluoroquinolones (FQs) are commonly used to treat urinary tract infections (UTIs), but some studies have suggested they may increase the risk of aortic aneurysm or dissection (AA/AD). However, no large-scale international study has thoroughly assessed this risk.

**Methods:**

A retrospective cohort study was conducted using a large, distributed network analysis across 14 databases from 5 countries (United States, South Korea, Japan, Taiwan, and Australia). The study included 13,588,837 patients aged 35 or older who initiated systemic fluoroquinolones (FQs) or comparable antibiotics (trimethoprim with or without sulfamethoxazole [TMP] or cephalosporins [CPHs]) for UTI treatment in the outpatient setting between JAN 01, 2010 and DEC 31, 2019. Patients were included if at the index date they had at least 365 days of prior observation and were not hospitalised for any reason on or within 7 days prior to the index date. The primary outcome was AA/AD occurrence within 60 days of exposure, with secondary outcomes examining AA and AD separately. Cox proportional hazards models with 1:1 propensity score (PS) matching were used to estimate the risk, with results calibrated using negative control outcomes. Analyses were subjected to pre-defined study diagnostics, and only those passing all diagnostics were reported. Hazard ratios (HRs) were pooled using Bayesian random-effects meta-analysis.

**Findings:**

Among analyses that passed diagnostics there were 1,954,798 and 1,195,962 propensity-matched pairs for the FQ versus TMP and FQ versus CPH comparisons respectively. For the 60-day follow-up there was no difference in risk of AA/AD between FQ and TMP (absolute rate difference [ARD], 0.21 per 1000 person-year; calibrated HR, 0.91 [95% CI 0.73–1.10]). There was no significant difference in risk for FQ versus CPH (ARD, 0.11 per 1000 person-year; calibrated HR, 1.01 [95% CI 0.82–1.25]).

**Interpretation:**

This large-scale study used a rigorous design with objective diagnostics to address bias and confounding. There was no increased risk of AA/AD associated with FQ compared to TMP or CPH in patients treated for UTI in the outpatient setting. As we only examined FQ used to treat UTIs in the outpatient setting, the results may not be generalisable to other indications with different severity.

**Funding:**

10.13039/501100008005Yonsei University College of Medicine, Government-wide R&D Fund project for infectious disease research (GFID), Republic of Korea, 10.13039/501100000925National Health and Medical Research Council (NHMRC) 10.13039/100015539Australian Government. 10.13039/100000738Department of Veterans Affairs (VA) Informatics and Computing Infrastructure (VINCI), 10.13039/100000738Department of Veterans Affairs, the United States Government.

**Research in context**

**Evidence before this study**

PubMed was searched for studies published in English prior to January 2024 using search terms fluoroquinolone, quinolone, ciprofloxacin, levofloxacin, aortic aneurysm, aortic dissection with search terms found in abstract and title or MESH headings. The reference lists of identified articles were also manually searched for additional relevant literature. Previous observational studies have identified that fluoroquinolones were associated with an increased risk of aortic aneurysm or dissection, and in response regulatory bodies have issued safety warnings and some have restricted use of fluroquinolones. Subsequent studies have reported contradictory results and suggest that risk of aortic aneurysm or dissection was related to the underlying condition being treated or surveillance bias, however there is still disagreement. There has been no international multi-database study that has assessed risk of aortic aneurysm or dissection following the use of fluoroquinolones using a consistent and best-practice study design to clarify this potential safety issue.

**Added value of this study**

This large-scale multinational multi-database study found that fluoroquinolones were not associated with an increased risk of aortic aneurysm or dissection compared to other common antibiotics in patients with urinary tract infections. These results do not support restricting fluoroquinolone use for urinary tract infections based on aortic risk, though other factors such as antibiotic resistance should still guide use.

**Implications of all the available evidence**

The findings of this study suggest that fluoroquinolones can be prescribed for urinary tract infections without consideration of risk of aortic aneurysm or aortic dissection despite contradictory evidence from previous studies and prior warnings. However, it is important to note that the risk of aortic aneurysm or dissection was measured up to 60 days, and this study only included patients treated for UTI in the outpatient setting, therefore the results may not be generalisable to other indications or different severities.

## Introduction

Fluoroquinolone (FQ) antibiotics are broad-spectrum antibiotics that treat a variety of infections, including urinary tract infections (UTIs). Although FQs are generally well-tolerated, a recent meta-analysis based on five observational post-market studies concluded that FQ use doubled the risk of incident aortic diseases.^1^ While aortic aneurysm (AA) and aortic dissections (AD) are rare, they are fatal in 65–90% of cases, especially when an AA ruptures.^2^

In response to safety concerns raised by observational studies and adverse events reported to regulatory bodies internationally, warnings were issued regarding the risk of AA/AD with FQ antibiotics.^3^, ^4^, ^5^ Studies have shown that the use of outpatient FQs has decreased following these safety warnings in some but not all settings, with the United Kingdom Medicines and Healthcare products Regulatory Agency recently issuing further restrictions on prescribing.^6^^,^^7^

Recent studies have suggested that the previous studies reporting associations between FQs and some adverse drug reactions (ADRs) may have been affected by indication bias or surveillance bias.^8^^,^^9^ A meta-analysis of the four studies listed in the United States of America (US) Federal Drug Administration (FDA) Drug Safety Communication regarding the association of FQs with AA/AD noted that confounding by indication may have influenced results and only one study included an active comparator.^10^

Given the conflicting results of previous studies, the recognised efficacy of FQs, and the serious nature of aortic aneurysm/aortic dissection (AA/AD) complications, we conducted a large-scale distributed network study to estimate the risk of AA/AD after FQ exposure for treatment of UTI compared to other antibiotics. We employed best-practice methodology to minimise potential systematic bias and used objective diagnostics to evaluate the analytical method performance.^11^

## Methods

### Data sources and procedures

This retrospective large-scale cohort study employed a distributed network approach across the Observational Health Data Science and Informatics (OHDSI) data network.^12^ The study protocol and analytic code were defined by May 2, 2023. Analyses from the 14 included data sources, that had been standardized to the Observational Medical Outcomes Partnership (OMOP) common data model (CDM) version 5, were completed by August 16, 2023. Data sources included 4 administrative claims (Optum's Clinformatics® Data Mart Database, IBM Health MarketScan® Commercial Claims and Encounters Database, IBM Health MarketScan® Multi-State Medicaid Database, and PharMetrics Plus) and 3 electronic health records (EHRs) (Columbia University Irving Medical Center data warehouse, Optum© de-identified Electronic Health Record Dataset, and Department of Veterans Affairs) from the US, 4 administrative claims (National Health Insurance Service-National Sample Cohort [Korea], Japan Medical Data Center [Japan], Japan Claims [Japan], and Longitudinal Patient Database in Australia [Australia]) and 3 EHRs (Taipei Medical University Clinical Research Database [Taiwan], Ajou University School of Medicine [Korea], and Yonsei University Health System [Korea]) from outside the US. Details of the included data sources can be found in Supplementary Method 1. All participating sites and data partners obtained approval or exemption from their institutional review boards prior to participating in the study. Due to the retrospective nature of the study, the requirement for informed consent was waived.

### Study design

The study utilised data from 1 January 2010 to 31 December 2019. The study period was restricted to the end of 2019 to avoid the potential impact of the COVID-19 pandemic on changes in antibiotic use. We performed a new-user cohort study to estimate the comparative risk of AA/AD with new use of FQs compared to alternative antibiotics. Graphical overview of the study design and included antibiotics is described in Supplementary Method 2. A comparison between the design of this study and the previously published studies is described in Supplementary Method 3.

### Participants

Patients were included at the date of first use of systemic FQs, trimethoprim with or without sulfamethoxazole (TMP), or cephalosporins (CPHs). The index date was defined as the day of initiating FQs, TMP, or CPHs for treatment of UTI. UTI was defined using the Systematised Nomenclature of Medicine Clinical Terms (SNOMED CT) concept Urinary tract infectious disease (68,566,005) and its descendant concepts, which include cystitis and pyelonephritis. Patients were included if at the index date they were aged 35 years or older, had at least 365 days of prior observation in the database, had a recorded condition occurrence of UTI on or within 7 days, and were not hospitalised for any reason on or within 7 days prior to the index date.

Three exposure cohorts were generated: (1) FQ exposure, (2) TMP exposure, or (3) CPH exposure. Only systemic exposure from medications administered orally or via injection were included in the exposure definitions. TMP and CPH were selected as comparator antibiotics as they are generally recommended in UTI treatment guidelines for both upper and lower UTIs, similarly to FQs.^13^^,^^14^ A full list of medicines included in each of these cohorts is provided in Supplementary Method 2.

To minimise confounding by indication we restricted the analysis to patients treated in an outpatient setting for UTIs, which are commonly localised, well-characterised infections with a similar severity profile across presentations.

### Procedures and outcomes

The primary outcome was diagnosis of either AA or AD during a hospital or emergency department visit in the 60 days after index date. The secondary endpoints included the occurrence of each outcome separately. To validate the outcome definition used in this study, a manual chart review was conducted using data from a tertiary hospital in Korea (Severance Hospital) following approval from the institutional review board (No. 4-2023-0261). Due to the retrospective nature of the study, the requirement for informed consent was waived. Briefly, a total of 100 cases each of AA and AD were randomly selected based on the same outcome definition of AA/AD used in this study, covering the period from January 1, 2010, to December 31, 2019. A manual chart review was performed to confirm whether the cases identified under this outcome definition were indeed diagnosed as AA or AD, and the positive predictive value was calculated. Patients were excluded if they had a recorded outcome event in the 365 days prior to index date. Details of the outcome definitions and the results of outcome validation are provided in Supplementary Method 4.

A time at risk window of 60 days after treatment initiation was employed for the primary analysis consistent with previous studies.^8^^,^^9^^,^^15^, ^16^, ^17^, ^18^

### Statistical analysis

Cox proportional hazards was used to estimate the hazard ratio of each outcome for each time at risk window in each data source after propensity score (PS) matching. To derive the PS, we used regularised regression including over 10,000 covariates characterising patient demographics, medical history, drug exposures, historical procedures, device exposures, and health service-use behaviors over 30 and 365 days before index date. All variables except race and ethnicity were binary (yes/no), with missing values assumed to indicate absence. Separate PS models were constructed for each comparator. Patients in the target cohort (FQs) were matched to the comparator cohorts (either TMP or CPH) using 1:1 matching on the PS.^19^

To identify the presence of systematic bias or unmeasured confounding, 50 negative control outcomes were selected (Supplementary Method 5). Hazard ratio (HR) estimates for each negative control outcome were generated using the same exposure cohorts as described above. This allowed for estimation of an empirical null distribution which was used to calibrate all HR estimates and p-values.^20^

A Bayesian random-effects meta-analysis was conducted to combine each site's HR estimate into a single aggregated HR using non-normal likelihood approximations to avoid bias due to small or zero counts.^21^

### Pre-defined study diagnostics

Three pre-defined objective study diagnostics were used to determine whether each analysis was valid. These included 1) assessment of patient characteristics to determine *covariate balance* between cohorts before and after PS adjustment, 2) preference score distributions to evaluate *empirical equipoise* and 3) expected absolute systematic error (EASE) to evaluate *residual bias*. Details of these diagnostics can be found in Supplementary Method 6. First, *covariate balance* was assessed using standardised difference of the mean (SMD) between FQ and comparator cohorts (either TMP or CPH). Sufficient covariate balance was achieved if SMD was less than 0.1 for all pre-defined covariates. Second, after application of PS matching, evaluation of *clinical equipoise* was assessed by calculation of the proportion of the overlap of the distribution of preference scores between the FQ and comparator cohorts. We defined equipoise to be sufficient if greater than 20% of patients in both cohorts had preference scores between 0.3 and 0.7.^22^ Third, *systematic error* was assessed using the EASE score.^20^ EASE was calculated by first fitting the systematic error distribution across the set of negative control outcomes and then taking the absolute expected value of the distribution. Lower EASE scores indicate little to no systematic error or unmeasured confounding. We considered that an EASE score less than 0.25 was adequate to indicate limited systematic bias. Only those analyses that passed all three diagnostics were included in the meta-analysis.

### Sensitivity analyses

Sensitivity analyses were performed by varying the follow-up risk windows as 30, 90 or 365-days after index date. The study protocol and executable analytic code are available online (https://github.com/ohdsi-studies/FluoroquinoloneAorticAneurysm). The research has been documented in compliance with the Strengthening the Reporting of Observational studies in Epidemiology (STROBE) guideline.^23^

### Patient and public involvement

No patients or members of the public were directly involved in the design or analysis of the reported data.

### Role of the funding source

The funder of the study had no role in study design, data collection, data analysis, data interpretation, or writing of the report. JLJ and SCY had full access to all the data in the study and takes responsibility for the integrity of the data and the accuracy of the data analysis. SCY had final responsibility for the decision to submit for publication.

## Results

### Participants and descriptive data

**Fig. 1:**
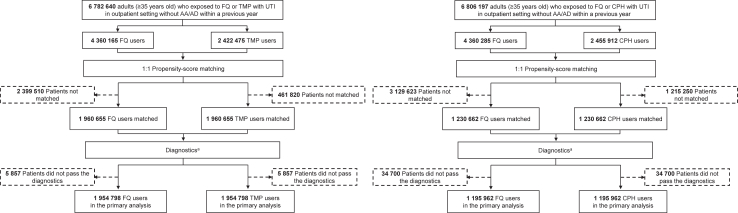
**Study flowchart of patients initiating fluoroquinolones, trimethoprim with or without sulfamethoxazole, or cephalosporins for urinary tract infection**. ^a^The objective study diagnostics included 1) assessment of patient characteristics to determine covariate balance between cohorts before and after propensity score adjustment, 2) preference score distributions to evaluate empirical equipoise, and 3) expected absolute systematic error to evaluate residual bias. All three diagnostic criteria needed to be satisfied for the analysis to be considered valid and included in the primary analysis. Abbreviations: FQ, fluoroquinolone; TMP, trimethoprim with or without sulfamethoxazole; CPH, cephalosporin; UTI, urinary tract infection; AA, aortic aneurysm; AD, aortic dissection.

A total of 14 databases were included in the study. Seven databases were from the United States, three were from South Korea, two were from Japan, one from Taiwan, and one from Australia. Before PS matching the cohorts in the FQs compared to TMP comparison included 6,782,640 patients (4,360,165 in the FQ cohort and 2,422,475 in the TMP cohort), and for the comparison between FQs and CPHs there were a total of 6,806,197 patients (4,360,285 in the FQ cohort and 2,455,912 in the CPH cohort) from 14 databases. After PS matching, there were 3,921,310 patients and 2,461,324 patients included in each analysis (Fig. 1).

The positive predictive values derived from the manual chart review were 97% for AA and 79% for AD, respectively.

### Main results or outcome data

#### Results from objective study diagnostics

Results from objective study diagnostics are presented in Supplementary Table S1. Selected baseline characteristics of patients in the FQ and TMP cohorts before and after PS matching in the Optum® EHR dataset are presented in Table 1. After PS matching, the absolute standardised mean differences (SMDs) for the 73,072 baseline covariates in the FQs versus TMP analysis and the 78,204 baseline covariates in the FQ versus CPH analysis were less than 0.1. There were 7 databases where the absolute SMDs of all predefined covariates were less than 0.1 between the FQ and TMP cohorts. There were 12 databases where the absolute SMDs of all predefined covariates were less than 0.1 between FQ and CPH cohorts. SMDs for all cohorts are presented in Supplementary Tables S2–S17.

**Table 1:** Baseline characteristics of patients in the Optum® EHR between FQ users and TMP users before and after propensity score matching.

Characteristic	Before propensity score matching			After propensity score matching		
	FQ (n = 1,093,755)	TMP (n = 620,448)	SDM	FQ (n = 478,507)	TMP (n = 478,507)	SDM
Age group, %^a^						
35–39	7.6	9.6	−0.07	9.6	10.4	−0.03
40–44	8.0	9.2	−0.04	9.1	9.5	−0.01
45–49	8.6	9.5	−0.03	9.6	9.8	−0.01
50–54	9.8	10.3	−0.01	10.2	10.5	−0.01
55–59	10.6	10.8	−0.01	10.8	11.0	0.00
60–64	10.1	10.0	0.00	9.9	10.0	0.00
65–69	9.7	9.3	0.02	9.4	9.3	0.00
70–74	9.3	8.6	0.02	8.6	8.3	0.01
75–79	12.6	10.7	0.06	10.7	10.3	0.01
80–84	12.3	10.5	0.06	10.5	9.5	0.03
85–89	1.3	1.6	−0.02	1.5	1.4	0.01
Sex: women, %						
Female	79.3	84.2	−0.13	84.2	84.8	−0.02
Male	20.7	15.8	−0.13	15.8	15.2	−0.02
Race, %^b^						
Asian	1.3	1.3	0.00	1.3	1.3	0.00
Black or African American	9.1	9.6	−0.02	9.5	9.7	−0.01
White	84.8	84.4	0.01	84.4	84.1	0.01
Ethnicity, %^c^						
Hispanic or Latino	4.6	4.4	0.01	4.3	4.4	−0.01
Not Hispanic or Latino	89.9	90.5	−0.02	90.4	90.3	0.00
Medical history, %^d^						
Hypertensive disorder	49.4	45.7	0.07	45.2	43.8	0.03
Atrial fibrillation	7.9	6.8	0.04	6.6	6.1	0.02
Heart failure	7.4	6.0	0.06	5.8	5.3	0.03
Ischemic heart disease	6.2	5.3	0.04	5.1	4.7	0.02
Peripheral vascular disease	4.8	4.1	0.03	4.1	3.6	0.03
Heart valve disorder	6.6	5.8	0.04	5.8	5.2	0.02
Cerebrovascular disease	5.3	4.6	0.03	4.5	4.2	0.01
Diabetes mellitus	21.9	19.9	0.05	19.6	18.7	0.02
Hyperlipidemia	42.3	39.7	0.05	39.4	38.1	0.03
Chronic liver disease	2.4	2.1	0.02	2.1	2.0	0.01
Renal impairment	13.2	10.7	0.07	10.2	9.3	0.03
Chronic obstructive lung disease	9.3	8.2	0.04	8.2	7.6	0.02
Crohn's disease	0.6	0.5	0.01	0.5	0.5	0.00
Ulcerative colitis	0.4	0.4	0.01	0.4	0.4	0.01
Dementia	5.0	4.2	0.04	4.1	3.8	0.01
Depressive disorder	18.7	19.5	−0.02	19.2	18.4	0.02
Human immunodeficiency virus infection	0.2	0.2	0.00	0.2	0.2	0.00
Psoriasis	1.0	0.9	0.01	0.9	0.9	0.01
Rheumatoid arthritis	2.1	1.8	0.02	1.8	1.6	0.01
Malignant neoplastic disease	11.2	10.2	0.03	10.1	9.4	0.02
Medication use, %^e^						
Antithrombotic agents	38.7	35.3	0.07	34.8	33.2	0.04
Agents acting on the renin-angiotensin system	35.0	31.8	0.07	31.4	30.4	0.02
Beta blocking agents	31.9	29.3	0.06	28.9	27.6	0.03
Calcium channel blockers	19.2	17.1	0.05	17.0	16.2	0.02
Diuretics	32.2	29.7	0.05	29.3	28.3	0.02
Drugs used in diabetes	21.7	20.0	0.04	19.7	18.7	0.03
Lipid modifying agents	37.4	35.0	0.05	34.4	33.3	0.02
Antiinflammatory and antirheumatic products	55.5	54.3	0.02	53.6	52.4	0.02
Immunosuppressants	3.7	3.7	0.00	3.4	3.2	0.01
Antidepressants	34.3	35.4	−0.02	34.9	34.0	0.02
Charlson comorbidity index^f^	2.1	1.9	0.06	1.9	1.7	0.05

To account for baseline differences between the two groups, PS-based matching was used. PSs were calculated in each database independently, based on available demographic characteristics, as well as the medical, medication, procedure exposure history, and health service-use behaviors of each database. More detailed balance data before and after PS adjustment can be explored at: https://data.ohdsi.org/FluoroquinoloneAorticAneurysm/.

Abbreviation: FQs, fluoroquinolones; TMP, trimethoprim with or without sulfamethoxazole; SDM, standardized difference of means; Optum® EHR, Optum® Electronic Health Records.

a Age groups over 90 were omitted.

b The race is reported based on the captured information in the database allowing missing values.

c The ethnicity is reported based on the captured information in the database allowing missing values.

d Medical history was identified by coded medical diagnosis within 1 year prior to the cohort entry.

e Medication use was identified by medication records within 1 year prior to the cohort entry. Both ATC class-level and ingredient-level drug uses were used to fit the PS model. The only class-level balances of drugs before and after PS matching is reported in this table.

f Charlson comorbidity (Romano adaptation) was calculated based on the medical history prior to the cohort entry.

**Fig. 2:**
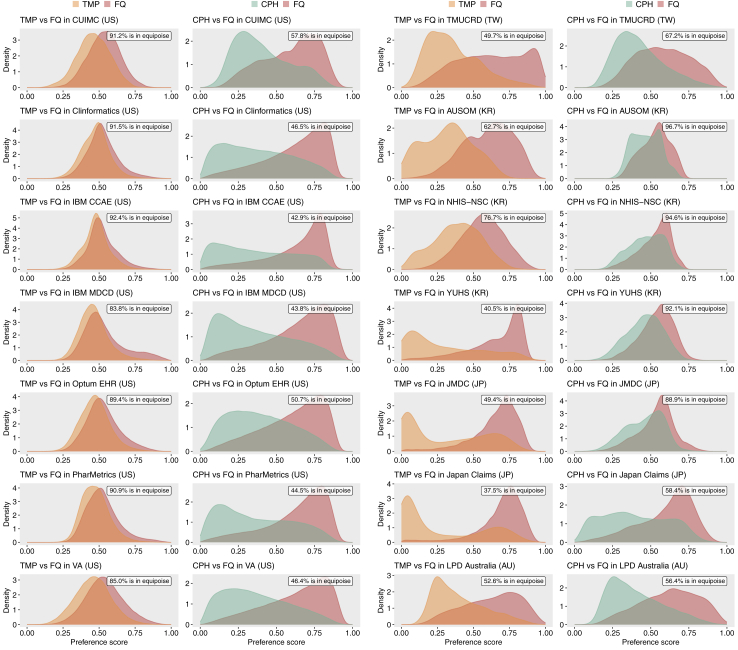
**Distribution of preference scores in target and comparator cohorts for the treatment of urinary tract infections in US and non-US databases**. The preference score distributions are shown for the target cohort (fluoroquinolone [FQ]) and comparator cohorts (trimethoprim with or without sulfamethoxazole [TMP] or cephalosporin [CPH]) before propensity score matching across the participating databases. The proportion of patients with preference scores between 0.3 and 0.7, indicating the empirical equipoise, is reported in parentheses for each comparison. A higher overlap in the preference score distributions suggests a greater proportion of patients in both cohorts who were equally likely to receive either treatment, thus indicating a higher level of equipoise. Abbreviations: FQ, fluoroquinolone; TMP, trimethoprim with or without sulfamethoxazole; CPH, cephalosporin; CUIMC, Columbia University Irving Medical Center data warehouse; Clinformatics, Optum's Clinformatics® Data Mart Database; IBM CCAE, IBM Health MarketScan® Commercial Claims and Encounters Database; IBM MDCD, IBM Health MarketScan® Multi-State Medicaid Database; Optum® EHR, Optum© de-identified Electronic Health Record Dataset; PharMetrics, PharMetrics Plus; VA, Department of Veterans Affairs; TMUCRD, Taipei Medical University Clinical Research Database; AUSOM, Ajou University School of Medicine; NHIS-NSC, National Health Insurance Service-National Sample Cohort; YUHS, Yonsei University Health System; JMDC, Japan Medical Data Center; LPD Australia, Longitudinal Patient Database in Australia

Preference score distribution overlap (clinical equipoise) exceeded 20% in all databases and for both comparisons (Fig. 2). The range of preference score overlaps was 0.85 to 0.92 for the comparison between FQs and TMP, and 0.43 to 0.58 for the comparison between FQs and CPH in data sources from US. For non-US countries, the range of preference score overlap was 0.38–0.77 for FQs v TMP and 0.56–0.97 for FQs versus CPH (Fig. 2).

The range of EASE, representing systematic bias, was 0.03–0.15 in each of the 7 US databases for FQs compared to TMP comparison. None of results from non-US databases passed the EASE diagnostic, suggesting a high level of systematic bias in the analyses from these databases. Among the comparison between FQs and CPH, the EASE values were less than 0.25 from the 7 US databases and 2 Asian databases.

Overall, there were 7 databases where all 3 study diagnostics were passed for the comparison of FQs with TMP and 9 databases where all 3 study diagnostics were passed for the comparison of FQs with CPH. Only those databases that passed study diagnostics were included in the analyses.

#### Association between FQs and active comparators and the risk of AA/AD within 60 days

**Fig. 3:**
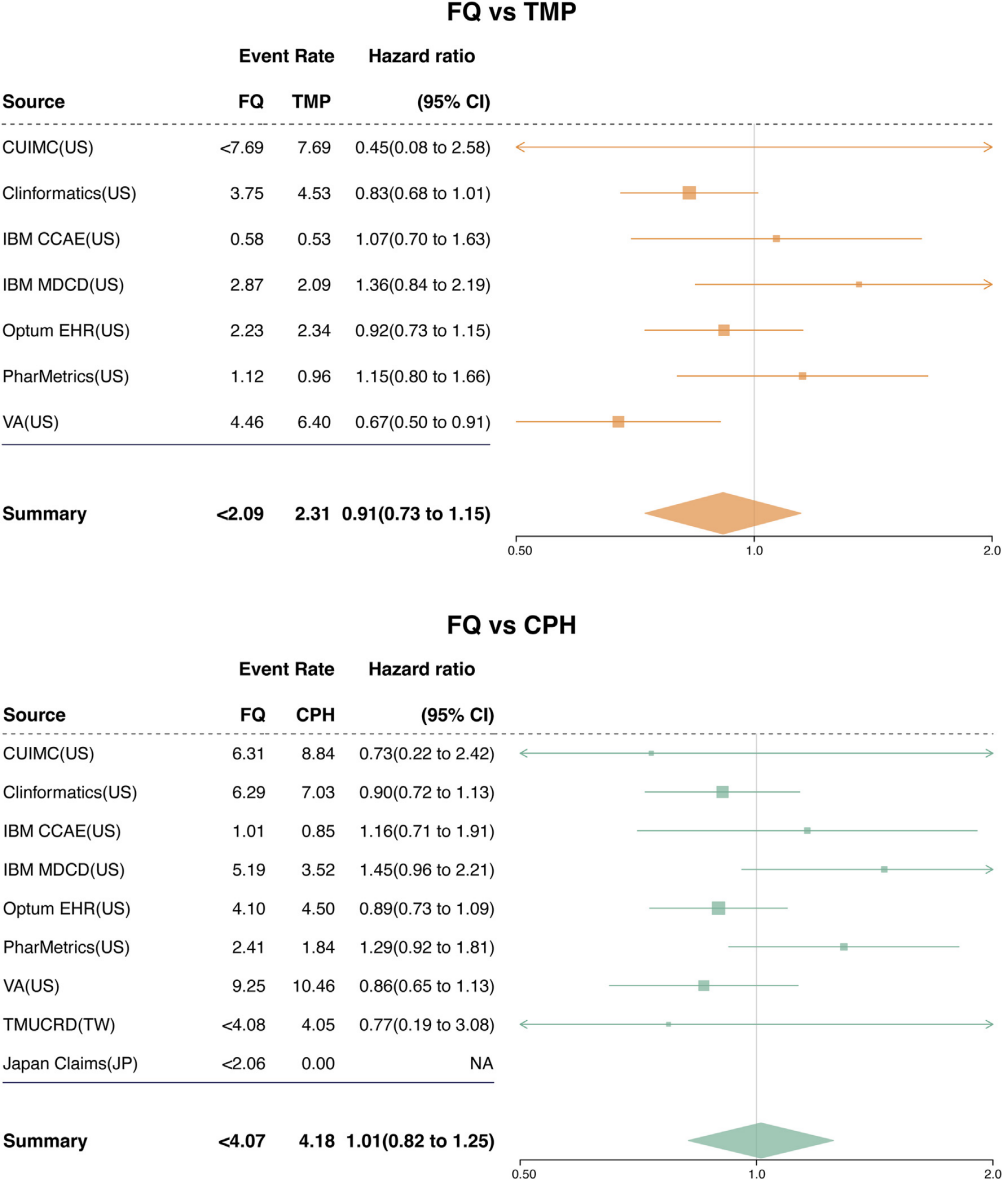
**Meta-analytic comparative risk of aortic aneurysm or dissection within 60 days after treatment initiation for urinary tract infection**. The forest plots show the calibrated hazard ratios (HRs) and 95% confidence intervals (CIs) for the risk of aortic aneurysm or dissection within 60 days after treatment initiation with fluoroquinolones (FQs) compared to trimethoprim with or without sulfamethoxazole (TMP) or cephalosporins (CPHs) for urinary tract infection. The event rates per 1000 person-years for each treatment group are also provided. In databases where the number of outcome events was less than 5, the exact count was not collected to protect patient privacy. The HRs were estimated using Cox proportional hazards models after propensity score matching in each database. The HRs were then calibrated based on the empirical null distribution derived from negative control outcomes to account for systematic bias. The calibrated HRs from each database were pooled using a Bayesian random-effects meta-analysis with non-normal likelihood approximations. The size of the data marker indicates the weight of the study. Error bars indicate 95% CIs. Abbreviations: FQ, fluoroquinolone; TMP, trimethoprim with or without sulfamethoxazole; CPH, cephalosporin; CI, confidence interval; CUIMC, Columbia University Irving Medical Center data warehouse; Clinformatics, Optum's Clinformatics® Data Mart Database; IBM CCAE, IBM Health MarketScan® Commercial Claims and Encounters Database; IBM MDCD, IBM Health MarketScan® Multi-State Medicaid Database; Optum® EHR, Optum© de-identified Electronic Health Record Dataset; PharMetrics, PharMetrics Plus; VA, Department of Veterans Affairs; TMUCRD, Taipei Medical University Clinical Research Database; NA, not applicable.

The incidence of the primary outcome after PS matching in each database is presented in Supplementary Table S18. The cumulative incidence of primary outcome at 60 days in each database is depicted in Supplementary Figure S1. In the meta-analysis, the 60-day AA/AD risk was not significantly different between FQ and TMP (absolute rate difference [ARD], 0.21 per 1000 person-year; calibrated HR, 0.91 [95% CI 0.73–1.15]; Fig. 3). Similarly, the 60-day AA/AD risk was not significantly different between FQs and CPHs, with a calibrated HR of 1.01 [95% CI 0.82–1.25] and an ARD of 0.11 per 1000 person-year. Among 50 negative control outcomes, 48 (96%) for FQs versus TMP and 47 (94%) for FQs versus CPH had meta-analytic HRs with 95% CIs covering 1 after calibration, suggesting minimal systematic bias (Supplementary Figure S2).

### Sensitivity analyses

The meta-analytic comparative risks for the individual outcomes of AA and AD are presented in Supplementary Figure S3. At 60 days, there was no difference in the risk of AA or AD when comparing FQ with TMP, as all 95% confidence intervals included 1. In the comparison of FQ versus CPH, the risk of AD was lower in FQ group (calibrated HR 0.63 [95% CI 0.42–0.94]), while the risk of AA did not differ (calibrated HR 1.07 [95% CI 0.83–1.38]).

The results of the sensitivity analyses with varying time windows and for each outcome separately are shown in Supplementary Figure S4. There was no significant difference in the risk of AA/AD between FQs and active comparators for any of the observation periods of 30, 90, and 365 days. When each outcome was considered separately, there was no significant difference in the risk of AA with FQs compared to the active comparators for any of the follow-up risk windows. For AD, FQ use was associated with a lower risk at 90 days compared to TMP and a lower risk at 60 and 90 days compared to CPHs.

## Discussion

In this large-scale observational multi-database distributed network study, FQs were not associated with an increased risk of AA/AD compared to other common antibiotics in patients with UTIs. The results were consistent across varied time windows of follow-up. This study is, to our knowledge, the largest study to provide evidence about the association of FQs use and the risk of AA/AD.

The risk of AA/AD is biologically plausible as exposure to FQs has been shown to stimulate matrix metalloproteinases provoking breakdown of collagen in a mouse model, and this breakdown of collagen has been found to be associated with risk of AD.^24^ From 2015 to 2018, four observational studies were published suggesting a positive association between FQ exposure and increased risk of AA/AD using nationwide population data sources.^15^, ^16^, ^17^^,^^25^ However, the studies were performed in different study populations and only one study employed an active comparator with another antibiotic, amoxicillin, in an attempt to reduce confounding.^15^, ^16^, ^17^^,^^25^ None of the studies restricted their cohorts by the clinical indication for antibiotic use, hence confounding by severity of indication is possible as FQs may be used in more severe infections and these infections may be associated with an increased risk of AA/AD. These studies^15^, ^16^, ^17^^,^^25^ all used different analytic approaches and results were not systematically assessed for potential bias. Studies published after the FDA warning have reported no association between FQ exposure and AA/AD after employing active comparators and addressing potential confounding, however, due to the low incidence of outcomes, these studies may have been underpowered.^8^^,^^9^^,^^18^^,^^26^ A more detailed comparison with previous studies can be found in Supplementary Method 3. While one study addressed systematic bias by employing two negative control outcomes,^9^ none of the studies assessed systematic bias rigorously by considering overlap in PS distributions (equipoise) or using a large set of negative control outcomes. In this study, the preference score overlaps between the cohorts for the treatment of UTI differed between US and non-US databases. This suggests that there are strong regional differences in antibiotic selection for treatment of UTIs. This may be influenced by antibiotic susceptibility patterns across countries which leads to variations in UTI treatment guidelines in each country.^13^^,^^14^^,^^27^ As the most appropriate comparator antibiotic may differ across countries and data sources, a multinational large-scale study was essential in allowing for rigorous results.

The main strength of this study was that we employed best-practice methodology to ensure transparency, reproducibility, and reliability of the results.^11^ We used a distributed network approach in which the detailed protocol standardised definitions of exposures, outcome measures, study design and approach to addressing confounding. Additionally, we used a standardised analytic package to generate the analytic results within each of the participating databases.^28^ The traceability of patient journeys was well-ensured through the use of both administrative claims and EHR databases containing longitudinal patient data, including several nationwide databases, along with the requirement for 365 days of prior observation and focus on outpatient UTI treatment creating a well-defined patient population. Our approach mirrored that of hypothetical randomised controlled trial by employing a new-user, active-comparator design with clearly defined indications and we implemented an outcome-agnostic, data-driven PS model, modelled using large-scale regularised regression. Moreover, we applied a suite of predefined objective diagnostics to ensure the quality of the evidence, focusing on sufficient covariate balance, overlap in preference score distributions, and minimal systematic error as evaluated by a comprehensive set of negative control outcomes. Only those analyses that passed all objective diagnostic tests were included in the meta-analysis.

The study has several limitations. Similar to prior studies, the definition of AA/AD relied on International Classification of Diseases, Ninth Revision, Clinical Modification (ICD-9-CM) and ICD-10-CM codes rather than imaging modalities, such as computed tomography, to diagnose the AA/AD. We were, however, able to validate our use of specific diagnosis codes in one Korean tertiary hospital. In that database, we found that, when compared to physician's manual chart review of discharge notes, the positive predictive value of the codes used were high (97% for AA (97/100) and 79% for AD (79/100)). Furthermore, the incidence of outcomes in this study is consistent with known incidence of abdominal aortic aneurysm (2.5 to 6.5 per 1000 person-years).^29^ As we only examined FQ used to treat UTIs in the outpatient setting, the results may not be generalisable to other indications with different severity. Antibiotics are also usually used for a relatively short period of treatment in patients with UTIs in the outpatient setting, therefore, the effects of long-term or cumulative use of FQs could not be evaluated. This study did not perform subgroup analysis by individual fluoroquinolone or cephalosporin ingredients due to the potential for drastically reduced statistical power and the variability in drug availability across countries, which may limit the generalisability of findings to specific drug types.

Regulatory warnings about the use of FQ and the risk of AA/AD may have affected the use of FQ as the time period for warnings differed across countries, however, the majority of the study period was before regulatory warnings about the risk of AA/AD.

The dose of FQs or other antibiotics was not considered in this study. Since the criteria for antibiotic dosage and duration are well established in UTIs being treated in the outpatient setting, it is likely that the variation in dose would be small. Some important risks, such as Marfan syndrome could not be considered separately. However, the proportion of patients with Marfan syndrome in our study population was less than 0.1% (Supplementary Table S19). Given the minimal prevalence of this condition, meaningful subgroup analysis or discussion of its role was not feasible. Most of the results passing diagnostics were derived from data sources in the US, so generalisability of results to other countries may be limited. As expected, the majority of the study population was female; however, consistent risk estimates were observed in the Department of Veterans Affairs database, which predominantly involved male patients.

In conclusion, this large-scale study used a rigorous design with objective diagnostics to reduce bias and confounding. There was no increased risk of AA/AD with FQ compared to TMP or CPH in patients treated for UTI in the outpatient setting. The results do not support restricting FQ use for the treatment of UTIs based solely on the potential risk of AA or AD.

## Contributors

JLJ and JHK contributed equally and are co-first authors. NP and SCY share senior authorship. JLJ, JHK, NP, and SCY developed the study protocol. EM developed definitions for outcomes. All authors contributed to the analysis of results and writing of the manuscript. JLJ and SCY had full access to and verify the underlying study data. SCY is the guarantor for this study. The corresponding author attests that all listed authors meet the authorship criteria and that others who met the criteria have not been omitted.

## Data sharing statement

The open, executable source code are publicly available online (https://github.com/ohdsi-studies/FluoroquinoloneAorticAneurysm). The study protocol is also available online (https://ohdsi-studies.github.io/FluoroquinoloneAorticAneurysm/Protocol.html). All results are publicly available at https://data.ohdsi.org/FluoroquinoloneAorticAneurysm with a dedicated web browser. The patient-level data were not shared due to concerns regarding patient privacy.

## Declaration of interests

All authors have completed the International Committee of Medical Journal Editors disclosure of interest form and declare the following disclosures. EM discloses previous consulting relationships with Janssen and Orpyx Medical Technologies, in areas unrelated to this work. DRM was supported by a Wellcome Trust Clinical Research Fellowship. RK received support from the National Heart, Lung, and Blood Institute of the National Institutes of Health (under award K23HL153775) and the Doris Duke Charitable Foundation (under award, 2022060). He also receives research support, through Yale, from BridgeBio, Bristol-Myers Squibb, and Novo Nordisk. He is a coinventor of U.S. Provisional Patent Applications 63/562,355, 63/177,117, 63/428,569, 63/346,610, 63/484,426, 63/508,315, 63/606,203, and 18/813,882, unrelated to current work. He is also a founder of Evidence2Health, a precision health platform to improve evidence-based cardiovascular care. SLD reports grants from Alnylam Pharmaceuticals, Inc., AstraZeneca Pharmaceuticals LP, Biodesix, Inc, Celgene Corporation, Cerner Enviza, GSK PLC, IQVIA Janssen Pharmaceuticals, Inc., Novartis International AG, Parexel International Corporation through the University of Utah or Western Institute for Veteran Research outside the submitted work. KKCM reported receiving grants from the Hong Kong Research Grant Council, the CW Maplethorpe Fellowship, UK, National Institute for Health and Care Research, European Commission Framework Horizon 2020, Innovation and Technology Commission of the Government of the Hong Kong Special Administrative Region, and personal fees from IQVIA Ltd outside the submitted work. SS and MVZ are employees of IQVIA. JPG, PBR and MJS are employees and shareholders of Johnson & Johnson. MAS receives grants and contracts from the US National Institutes of Health, US Food & Drug Administration and Johnson & Johnson outside the scope of this work. MEM received grants from NIH NHLBI and VA ORD and has a patent pending for an AI algorithm related to surveillance. KKCM received grants from the CW Maplethorpe Fellowship, IQVIA, the European Union Horizon 2020, the UK National Institute of Health Research, the Hong Kong Research Grant Council, and the Hong Kong Innovation and Technology Commission. NP received a grant from NHMRC (GNT 1196900). SCY reports being a chief technology officer of PHI Digital Healthcare; and grants from Daiichi Sankyo.
