# Supplementary material for: Risk of aortic aneurysm or dissection following use of fluoroquinolones: a retrospective multinational network cohort study
Source: eClinicalMedicine. 2025 Feb 1;81:103096. doi: 10.1016/j.eclinm.2025.103096 (PMC11836508; doi:10.1016/j.eclinm.2025.103096)
Supplement: Supplementary Methods, Figures and Tables [file mmc1.pdf]

ONLINE SUPPLEMENT

Risk of aortic aneurysm or dissection following use of fluoroquinolones: multinational network cohort study

Jack L Janetzki et al.

Supplementary Online Content

Supplementary method 1. Data Source 2

Supplementary method 2. Overview of study design 4

Supplementary method 3. Comparison of study designs between previously published papers and this study 7

Supplementary method 4. Outcome definitions 14

Supplementary method 5. Negative control outcomes 18

Supplementary method 6. Study diagnostics for cohort study 20

Supplementary table 1. Results of pre-defined study diagnostics 22

Supplementary table 2. Baseline characteristics of patients in the CUIMC between FQ users and TMP users 23

Supplementary table. Baseline characteristics of patients in Clinformatics® between FQ users and TMP users 26

Supplementary table 4. Baseline characteristics of patients in the IBM CCAE between FQ users and TMP users 29

Supplementary table 5. Baseline characteristics of patients in the IBM MDCD between FQ users and TMP users 31

Supplementary table 6. Baseline characteristics of patients in the Optum® EHR between FQ users and TMP users 34

Supplementary table 7. Baseline characteristics of patients in the PharMetrics between FQ users and TMP users 37

Supplementary table 8. Baseline characteristics of patients in the VA between FQ users and TMP users 39

Supplementary table 9. Baseline characteristics of patients in the CUIMC between FQ users and CPH users 42

Supplementary table 10. Baseline characteristics of patients in the Clinformatics® between FQ users and CPH users 45

Supplementary table 11. Baseline characteristics of patients in the IBM CCAE between FQ users and CPH users 48

Supplementary table 12. Baseline characteristics of patients in the IBM MDCD between FQ users and CPH users 50

Supplementary table 13. Baseline characteristics of patients in the Optum® EHR between FQ users and CPH users 53

Supplementary table 14. Baseline characteristics of patients in the PharMetrics between FQ users and CPH users 56

Supplementary table 15. Baseline characteristics of patients in the VA between FQ users and CPH users 58

Supplementary table 16. Baseline characteristics of patients in the TMUCRD between FQ users and CPH users 61

Supplementary table 17. Baseline characteristics of patients in the Japan Claims between FQ users and CPH users 64

Supplementary table 18. Incidence rates of primary endpoints 66

Supplementary table 19. Prevalence of risk factors (Marfan's syndrome, Ehlers-Danlos syndrome, Coarctation of aorta, Turner syndrome and bicuspid aortic valve). 66

Supplementary figure 1. Kaplan-Meier plots for the risks of the primary outcome 68

Supplementary figure 2. Systematic error control of effect estimation in the meta-analysis 70

Supplementary figure 3. The risk of the secondary outcomes in fluoroquinolone vesus trimethoprim with or without sulfamethoxazole or fluoroquinolone versus cephalosprin 71

Supplementary figure 4. Hazard ratios (95% CI) across time at risk windows between FQ and TMP or FQ and CPH for each outcome separately and for the composite outcome (AA/AD) 73

Supplementary method 1. Data Source

The selection of these secondary data sources for the OHDSI data network study is based on their comprehensive population coverage, extensive historical data, diverse data capture processes, voluntary participation, and the availability of verified outcome information, ensuring robust and reliable research outcomes.

Data source, country	Population	Patients (millions)	History	Data Capture Process and Short Description
Columbia University Irving Medical Center data warehouse (CUMC)	USA, general	6	1985-2022	Non-profit academic medical center
Optum Clinformatics® Data Mart Database (Clinformatics®)	USA, general	95	2000-2022	Optum Clinformatics® Data Mart Database is derived from a database of administrative health claims for members of large commercial and Medicare Advantage health plans.The database includes approximately 17-19 million annual covered lives, for a total of over 65 million unique lives over a 12 year period (1/2007 through 12/2019). Clinformatics(R) Data Mart is statistically de-identified under the Expert Determination method consistent with HIPAA and managed according to Optum® customer data use agreements. CDM administrative claims submitted for payment by providers and pharmacies are verified, adjudicated and de-identified prior to inclusion. This data, including patient-level enrollment information, is derived from claims submitted for all medical and pharmacy health care services with information related to healthcare costs and resource utilisation. The population is geographically diverse, spanning all 50 states. Clinformatics® also provides date of death (month and year only) for members with both medical and pharmacy coverage from the Social Security Death Master File (however after 2011 reporting frequency changed due to changes in reporting requirements) and location information for patients is at the US state level.
IBM Health MarketScan Commercial Claims and Encounters Database (IBM CCAE)	USA, general	167	2000-2023	Adjudicated health insurance claims across the continuum of care (e.g., inpatient, outpatient, pharmacy) from large employers and health plans who provide private healthcare coverage to employees, their spouses and dependents
IBM Health MarketScan Multi-State Medicaid Database (IBM MDCD)	USA, general	35	2006-2022	Adjudicated health insurance claims for Medicaid enrollees from multiple states covered under fee-for-service and managed care plans and includes claims across the continuum of care (e.g., inpatient, outpatient, pharmacy).
Optum® de-identified Electronic Health Record dataset (Optum® EHR)	USA, general	108	2007-2023	Combined claims and electronic health record data derived from >7000 hospitals and >7000 clinics. Clinical information includes vital signs, immunisations, allergies, medications, diagnoses, procedures and other data some of which are derived using natural language processing on provider notes.
PharMetrics Plus (PharMetrics)	USA, general	170	2014-2022	Data is from 2006 - 2022 and comprises of fully adjudicated medical and pharmacy claims. It contains a longitudinal view of inpatient and outpatient services, prescription and office/outpatient administered drugs, costs and enrollment information. With IQVIA Adjudicated Health Plan Claims, an enrolled patient can be tracked across all sites of care: hospital, specialist, emergency room, pharmacy, primary care, and more.
Department of Veteran Affairs (VA)	USA, veterans, older, racially diverse	12	2000-study execution date	National VA healthcare system, the largest integrated provider of medical services in the USA, providing care at 170 VA medical centers and 1063 outpatients facilities

Taipei Medical University Clinical Research Database (TMUCRD)	Taiwan, general	3.8	1998-2020	Non-profit academic medical center
Ajou University School of Medicine (AUSOM)[1]	Korea, general	2.8	1994-2019	Non-profit academic medical center
Korean National Health Insurance System-National Sample Cohort (NHIS-NSC)[2]	Korea, general	1	2002-2013	the national administrative claims database covering the South Korea population. It contains a 2% population sample cohort
Yonsei University Health System (YUHS)	Korea, general	5.4	1997-2022	Non-profit academic medical center
Japan Medical Data Center (JMDC)	Japan, general	15	2005-2022	A payer based database that has collected claims, ledger of the insured people and health checkup results from more than 250 payers. It covers workers and their dependents aged under 74. All medical history of the insured people are available and patient reported outcome research can be done through payers on-demand basis. Those aged 66 or older are less representative as compared with whole population in the nation. When estimated among the people who are younger than 66 years old, the proportion of children younger than 18 years old in JMDC is approximately the same as the proportion in the whole nation.
Japan Claims	Japan, general	14	1950-2023	Claims data on Japanese data
Longitudinal Patient Database Australia (LPD Australia)	Australia, general	2.8	2013-2023	Australia EMR source data contains medical history, allergies, immunisations, contacts and related to them medical events (test results, diagnoses, biometrics, prescriptions)

Supplementary method 2. Overview of study design

Supplementary method 2-1. Graphical overview of the cohort study design

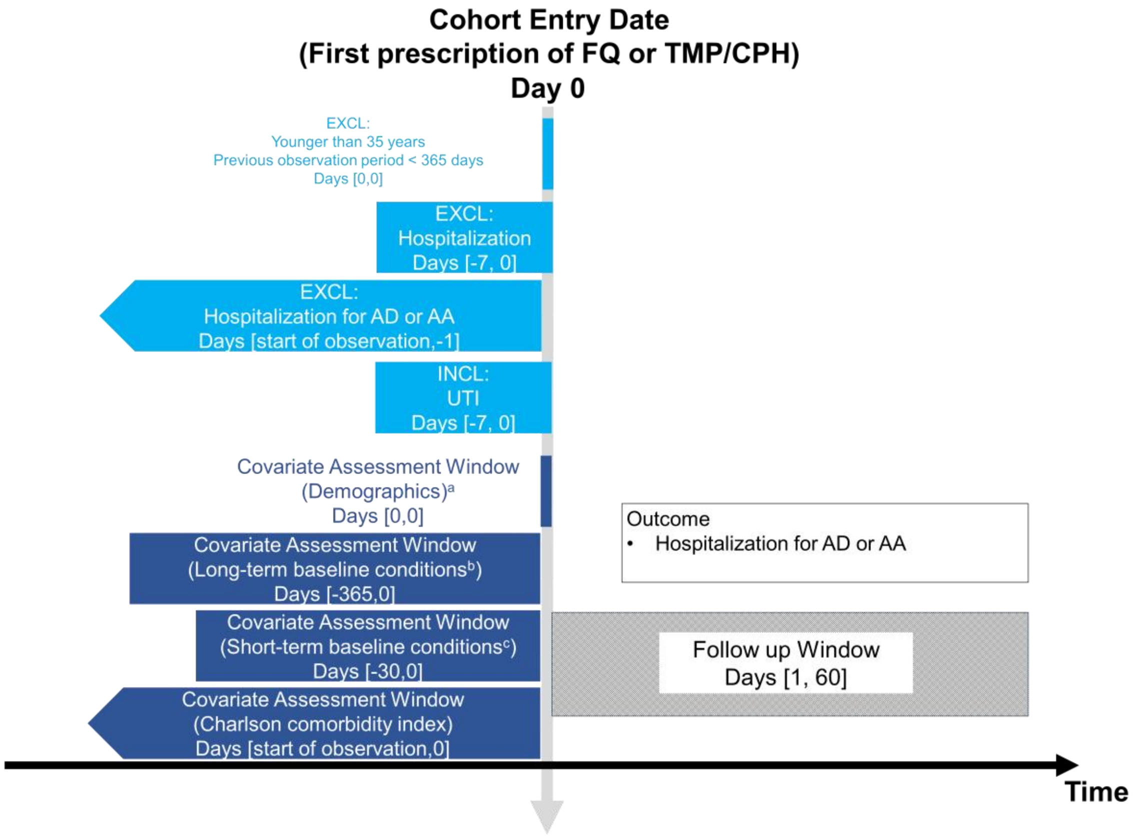

Note: (a) Age, sex, race, ethnicity, index year, index month; (b) Diagnosis, Drug exposure, Procedure, Device, and Measurement; (c) Diagnosis, Drug exposure, Procedure, Device, and Measurement;

Abbreviation: FQ, fluoroquinolone; TMP, trimethoprim with or without sulfamethoxazole; CPH, cephalosporin; UTI, urinary tract infection; AD, aortic dissection; AA, aortic aneurysm

Supplementary method 2-2. Included drug lists

To ensure systemic exposure, we excluded medications for otic or optic use only.

List of included fluoroquinolones:

Concept ID	Concept name
1721543	norfloxacin
35198003	pazufloxacin mesilate
1747032	grepafloxacin
19041153	temafloxacin
1592954	delafloxacin
1716721	gemifloxacin
923081	ofloxacin
19050750	fleroxacin
1712549	trovafloxacin
35197938	garenoxacin mesilate hydrate
36878831	nadifloxacin
40161662	besifloxacin
43009030	tosufloxacin tosylate hydrate
1743222	enoxacin
35834909	lascufloxacin hydrochloride
35198165	sitafloxacin hydrate
1789276	gatifloxacin
1797513	ciprofloxacin
1742253	levofloxacin
1716903	moxifloxacin
35197897	prulifloxacin
19027679	pefloxacin
1733765	sparfloxacin
1707800	lomefloxacin

List of included trimethoprim with or without sulfamethoxazole products:

Concept ID	Concept Name
40081374	sulfamethoxazole / trimethoprim Injectable Solution
36882762	sulfamethoxazole / Trimethoprim Injectable Suspension
40220482	sulfamethoxazole / trimethoprim Injection
35153537	Sulfamethoxazole / Trimethoprim Oral Granules
40147374	sulfamethoxazole / trimethoprim Oral Solution
40081379	sulfamethoxazole / trimethoprim Oral Suspension
40081388	sulfamethoxazole / trimethoprim Oral Tablet
1705674	trimethoprim

List of included cephalosporins:

Concept ID	Concept Name
40798709	cefacetrile
1796435	cefixime
40798704	cefmenoxime
19072255	cefmetazole
43008993	cefminox sodium
19028286	cefodizime
19072857	cefonicid
1773402	cefoperazone
19028288	ceforanide
1774470	cefotaxime
1774932	cefotetan
19051271	cefotiam
1775741	cefoxitin
43009045	cefpiramide sodium
1749008	cefpodoxime
1738366	cefprozil
43009083	cefroxadine
19051345	cefsulodin
1776684	ceftazidime
35198137	cefteram pivoxil
43008994	ceftezole sodium
1749083	ceftibuten
1777254	ceftizoxime
1777806	ceftriaxone
1778162	cefuroxime

1786621	cephalexin
19052683	cephaloridine
19086759	cephalothin
19086790	cephapirin
1786842	cephradine
43009087	flomoxef sodium
1708100	loracarbef
19126622	moxalactam
1768849	cefaclor
1769535	cefadroxil
19070174	cefamandole
19070680	cefatrizine
40798700	Cefazedone
1771162	cefazolin
43009082	cefbuperazone sodium
43009044	cefcapene pivoxil hydrochloride hydrate
1796458	cefdinir
1747005	cefditoren
19028241	cefetamet

Supplementary method 3. Comparison of study designs between previously published papers and this study

	Study design	Data sources	Indication	Active comparators	Rationale for selecting comparators	Equipoise assessment	Outcomes	Outcome definitions	Outcome PPV	Time window	Covariate balance	Systematic error control
This study	A retrospective cohort study	CUMC(US)<br>IBM CCAE (US)<br>IBM MDCD (US)<br>Clinformatics® (US)<br>Optum® EHR (US)<br>PharMetrics (US)<br>VA (US)<br>TMUCRD (TW)<br>AUSOM (KR)<br>NHIS-NSC (KR)<br>YUHS (KR)<br>JMDC (JP)<br>Japan Claims (JP)<br>LPD Australia (AU)	Urinary tract infections	Trimethoprim and sulfamethoxazole<br><br>Cephalosporin	Based on the recommendations of the treatment guidelines	Achieve equipoise in comparisons between fluoroquinolones and comparators.	Aortic aneurysm or aortic dissection	ICD-9 and ICD-10<br><br>Hospitalisation and emergency department visit	97% for AA, 79% for AD	60 days	Large-scale propensity score matching	Using a large set of negative controls

	Study design	Data sources	Indication	Active comparators	Rationale for selecting comparators	Equipoise assessment	Outcomes	Outcome definitions	Outcome PPV	Time window	Covariate balance	Systematic error control
JAMA Intern Med. 2015;175(11):1839-1847 [3]	A nested case-control study	Taiwan's National Health Insurance Research Database	N/A	N/A	N/A	N/A	Aortic aneurysm or aortic dissection	ICD-9-CM<br><br>Hospitalisation<br><br>The use of advanced imaging studies	92%	60 days	A propensity score for adjustment and matching	N/A
BMJ Open 2015;5:e010077.[4]	A retrospective cohort study	Ontario Registered Persons Database and Ontario Drug Benefits database	N/A	N/A	N/A	N/A	Aortic aneurysm	ICD-9 and ICD-10<br><br>Hospitalisation and emergency department visit	N/A	30 days	Cox proportional hazards model	Amoxicillin as negative tracer exposure<br><br>Clostridium difficile infection as positive tracer outcome

	Study design	Data sources	Indication	Active comparators	Rationale for selecting comparators	Equipoise assessment	Outcomes	Outcome definitions	Outcome PPV	Time window	Covariate balance	Systematic error control
BMJ 2018;360:k678[5]	A retrospective cohort study	Swedish National Prescribed Drug Register, National Patient Register, and Statistics Sweden	N/A	Amoxicillin	Approved indications largely overlap with those of fluoroquinolones	N/A	Aortic aneurysm or aortic dissection	ICD-10<br><br>Primary diagnosis<br><br>Hospitalisation or emergency department visit, or death	92-100%	60 days	A propensity score matched design	Sensitivity analysis
J Am Coll Cardiol. 2018 Sep, 72 (12) 1369-1378.[6]	A case-crossover study	Taiwan's National Health Insurance Research Database	N/A	N/A	N/A	N/A	Aortic aneurysm or aortic dissection	ICD-9-CM<br><br>Hospitalisation<br><br>Diagnostic evidence obtained by<br><br>advanced imaging studies	92%	60 days	The disease risk score-matched case-time-control analysis	N/A

	Study design	Data sources	Indication	Active comparators	Rationale for selecting comparators	Equipoise assessment	Outcomes	Outcome definitions	Outcome PPV	Time window	Covariate balance	Systematic error control
JAMA Intern Med. 2020;180(12):1587-1595[7]	A nested case-control study	Taiwan's National Health Insurance Research Database	Various sites of infections	Amoxicillin-clavulanate<br><br>Ampicillin-sulbactam<br><br>Extended-spectrum cephalosporins	Based on the recommendations of the treatment guidelines in Taiwan	N/A	Aortic aneurysm or aortic dissection	ICD-9<br><br>Hospitalisation and emergency department visit	89% to 100% for AA and 78% to 92% for AD	60 days	Adjusted for matching factors, baseline covariates, and concomitant antibiotic use	Achilles tendon rupture and any type of tendon rupture as positive contols
JAMA Intern Med. 2020;180(12):1596-1605[8]	A retrospective cohort study	US health insurance claims database (IBM MarketScan)	Pneumonia<br><br>Urinary tract infections	Azithromycin for pneumonia<br><br>Trimethoprim and sulfamethoxazole for urinary tract infections<br><br>Amoxicillin without indication for treatment	Clinically appropriate	N/A	Aortic aneurysm or aortic dissection	ICD-9-CM<br><br>Hospitalisation	85-92%	60 days	1:1 propensity score matching to control for 85 potential confounders	Hospitalization for heart failure and acute myocardial infarction as negative controls

	Study design	Data sources	Indication	Active comparators	Rationale for selecting comparators	Equipoise assessment	Outcomes	Outcome definitions	Outcome PPV	Time window	Covariate balance	Systematic error control
JAMA Surg. 2021;156(3): 264-272[9]	A retrospective cohort study	US health insurance claims database (IBM MarketScan)	Variuos sites of infections	Amoxicillin-clavulanate Azithromycin<br><br>Cephalexin<br><br>Clindamycin<br><br>Trimethoprim and sulfamethoxazole	Based on commonly prescribed antibiotics for similar indications	N/A	Aortic aneurysm or aortic dissection	ICD-9, ICD-10, and CPT	No report	90 days	Inverse probability of treatment weighting	Sensitivity analysis

	Study design	Data sources	Indication	Active comparators	Rationale for selecting comparators	Equipoise assessment	Outcomes	Outcome definitions	Outcome PPV	Time window	Covariate balance	Systematic error control
JAMA Cardiol. 2023;8(9):865-870.[10]	Cohort and case-crossover studies	2 databases of UK primary care records (Clinical Practice Research Datalink Aurum and GOLD primary care records)	N/A	Cephalosporins	Similar prescribing profile in UK practice to fluoroquinolones	N/A	Aortic aneurysm or aortic dissection	ICD-10<br><br>Hospitalisation	No report	60 days	Potential confounders were adjusted for using stabilized inverse probability of treatment weights with a propensity score estimated by logistic regression	A positive control outcome, tendon rupture

	Study design	Data sources	Indication	Active comparators	Rationale for selecting comparators	Equipoise assessment	Outcomes	Outcome definitions	Outcome PPV	Time window	Covariate balance	Systematic error control
Eur Heart J. 2023 Nov 7;44(42):4476-4484[11]	Cohort study and a self-controlled case series	National Health Insurance Service of the Republic of Korea	N/A	Third-generation cephalosporin	Broad antimicrobial spectrum, clinical indications, and favourable toxicity profile that matches fluoroquinolone	N/A	Aortic aneurysm or aortic dissection	ICD-10<br><br>Hospitalisation or in-hospital death	No report	1 year for Cox proportional hazard model	Inverse probability of treatment weighting for Cox proportional hazard model	Sensitivity analysis

Supplementary method 4. Outcome definitions

For each outcome, we developed an operational phenotype definition to determine if observational data could in fact support evaluation of the outcome. Where possible, concept sets originated with published code lists (eg ICD-9-CM, ICD-10, CPT-4, and ICD-9 procedure). We developed definition of outcome cohorts and query to extract them using ATLAS, the OHDSI open-source platform (https://github.com/OHDSI/atlas).

Outcome	ICD-9-CM	ICD-10	CPT4	ICD-9 procedure	PPV, % (n)[a]
Aortic aneurysm	441; 441.0; 441.1; 441.2; 441.3; 441.4; 441.5; 441.6; 441.7; 441.9[3,7–9]	I71.0; I71.1; I71.2; I71.3; I71.4; I71.5; I71.6; I71.8; I71.9[5,9–11]	0001T; 0002T; 0033T; 0034T; 0035T; 0036T; 0039T; 0078T; 0079T; 0080T; 0081T; 33720; 33877; 33880; 33881; 34800; 34802; 34803; 34804; 34805; 34813; 34830; 34831; 34832; 35081; 35082; 35091; 35092; 35102; 35103; 75952; 75953; 9001F; 9003F; 9004F[9]	39.71; 39.73	97 (97/100)
Aortic dissection	441.0; 441.00; 441.01; 441.02; 441.03[3,7–9]	I71.0[5,9–11]			79 (79/100)

[a] Positive Predictive Value (PPV) was calculated based on hospitalization or ER visits at any diagnosis position based on the ICD-10 code in the single Korean tertiary hospital, Severance Hospital (Yonsei University Health System).

The term ‘Aortic diseases’ has been used to collectively refer to non-ruptured aortic aneurysm (AA), aortic aneurysm rupture (AR), and aortic dissection (AD), among other pathologies that affect the aorta. Aortic diseases have become the subject of considerable contemporary study in observational data assets. Renewed focus has been brought to these entities in view of their possible association to fluoroquinolone (FQ) use [8,9,12–14], which have been associated to other collagen related side effects.

We undertook a review of existing phenotype definitions in observational studies examining fluoroquinolone exposure and Aortic diseases, See the table below.

	Disease	Hospitalization Required?	Primary position?	Clean window?	Definition	Database	Other
Son et al.[13]	AA, AR, AD	No	NA	365d	ICD10 I71.0-I71.9	NHIS, (Korea)	Age> 40; image codes in sensitivity analysis
Gopalakrishnan et al. [8]	AA, AR, AD	Yes	Yes	All time	ICD9 441 441.(0-7,9) 441.0(0-3)	IBM MarketScan (US)	Age >50
Newton et al. [9]	AA, AD, Iliac aneurysm, Other	No	NA	180d	ICD9, ICD10 (see reference)	IBM MarketScan (US)	Age 18-64
Pasternak et al.[14]	AA, AR, AD	Yes	Yes	All time	ICD10 I71.0-I71.9	Swedish registry (Sweden)	Age > 50
Dong et al.[7]	AA, AR, AD	Yes	No	All time	ICD-9 441.(0-7,9)	Taiwan NHIRD	Age ≥20

Lee et al.[3]	AA, AR, AD	Yes	No	All time	ICD9 441.(1-7,9) 441.0(0-3) AND imaging	Taiwan NHIRD	Age ≥ 18
Daneman et al.[4]	AA, AR, AD	Yes	Yes	365d	ICD9 441, ICD10 I710-I719, ICD9 441.(0-5), ICD10 I710-11,13, 15, 18)	Ontario Registered Persons Database (Canada)	Age ≥ 65
Lee et al. [6]	AA, AR, AD	Yes	No	All time	ICD-9CM 441.1-441.7, 441.9 or 441.0, 441.00-441.03) plus imaging	Taiwan Longitudinal Health Insurance Database	All patients

Characteristics of prior outcome definitions used in observational studies examining the impact of fluoroquinolones on the incidence of aortic disease (and arterial aneurysms at other sites). NHIRD - National Health Insurance Research Database

The table above illustrates some of the identified heterogeneity in previous modelling decisions for vascular aneurysm related disease outcomes. Among others, these include differences in disease site, requirement of hospitalisation, length of any 'clean' window prior to index, terminology and codes used, and the requirement that a diagnosis code be in 'primary' position. This latter requirement can serve to limit the generalisability of a definition depending on the provenance of the data and interpretation assigned to the 'primary' position of a claims record, which can vary in claims-based health records in different jurisdictions.

Methods

Consistent with prior studies in this area, we sought to define a composite outcome of Aortic disease as the outcome of interest in a safety study with fluoroquinolones. Using the SNOMED hierarchy and vocabulary mappings maintained by the OHDSI community, we created concept sets for Aortic Aneurysm (AA) (inclusive of AAR), and Aortic Dissection (AD).

Our cohort logic was implemented as follows:

1) Subjects enter the cohort based on a condition, observation, or procedure occurrence (of Aortic Disease related procedures) of one of those AA or AD concepts
2) We place further constraints on the record to restrict to patients who have the diagnosis made in an inpatient or emergency department visit.
3) Finally, to restrict to incident Aortic Disease, we require 365 days of prior observation in a database, with no prior condition, procedure, or observation occurrence of an AA or AD.

We then looked to perform a population-level validation of this cohort definition using CohortDiagnostics[15], a tool in the OHDSI software ecosystem. Population-level validation involves executing the cohort definition across several databases, and characterising the resulting cohort by examining attributes such as the incidence rate of cohort entry across time, and population level cohort features across different temporal windows relative to the cohort entry date. From these, the researcher can infer risk factors, diagnostic investigations, therapies, and complications associated with the cohort before, on the day of, and after the index date of cohort entry. The incidence of these covariates can be compared to expectations based on clinical experience and referential sources, to determine whether there are broad concerns with respect to cohort definition sensitivity (inferred by markedly lower incidence rates of disease than those suggested by other sources), specificity (inferred by seeing populations with characteristics not keeping with the disease of interest), or index date misclassification (inferred by seeing characteristics of the disease of interest before or well after the defined index date).

A composite outcome represents an additional challenge to population level validation in as much as several distinct clinical entities can be represented in the composite cohort, complicating comparisons against expected disease trajectories. To address this, we created distinct definitions for AA, AAR, and AD, and examined each of their population level attributes separately.

Results and Discussion

Characterization with CohortDiagnostics revealed that incidence rates of each of those aortic diseases where generally in line with literature sources, suggesting no major sensitivity error when using a single occurrence of a diagnostic code in any position.

With regards to specificity, examination of cohort features were felt to generally correspond to expectations. A broad sense of performance in this regard can be achieved by comparing the definition in question, to a definition expected to be more specific (achieved in this case by requiring additional conditional occurrences). The cohorts specified by the two definitions can be compared graphically on covariate plots in Cohort Diagnostics. The figure below displays covariate plots for AA, AAR and AD wherein the candidate definition (with one condition occurrence) is compared to a more specific definition (requiring two condition occurrences).

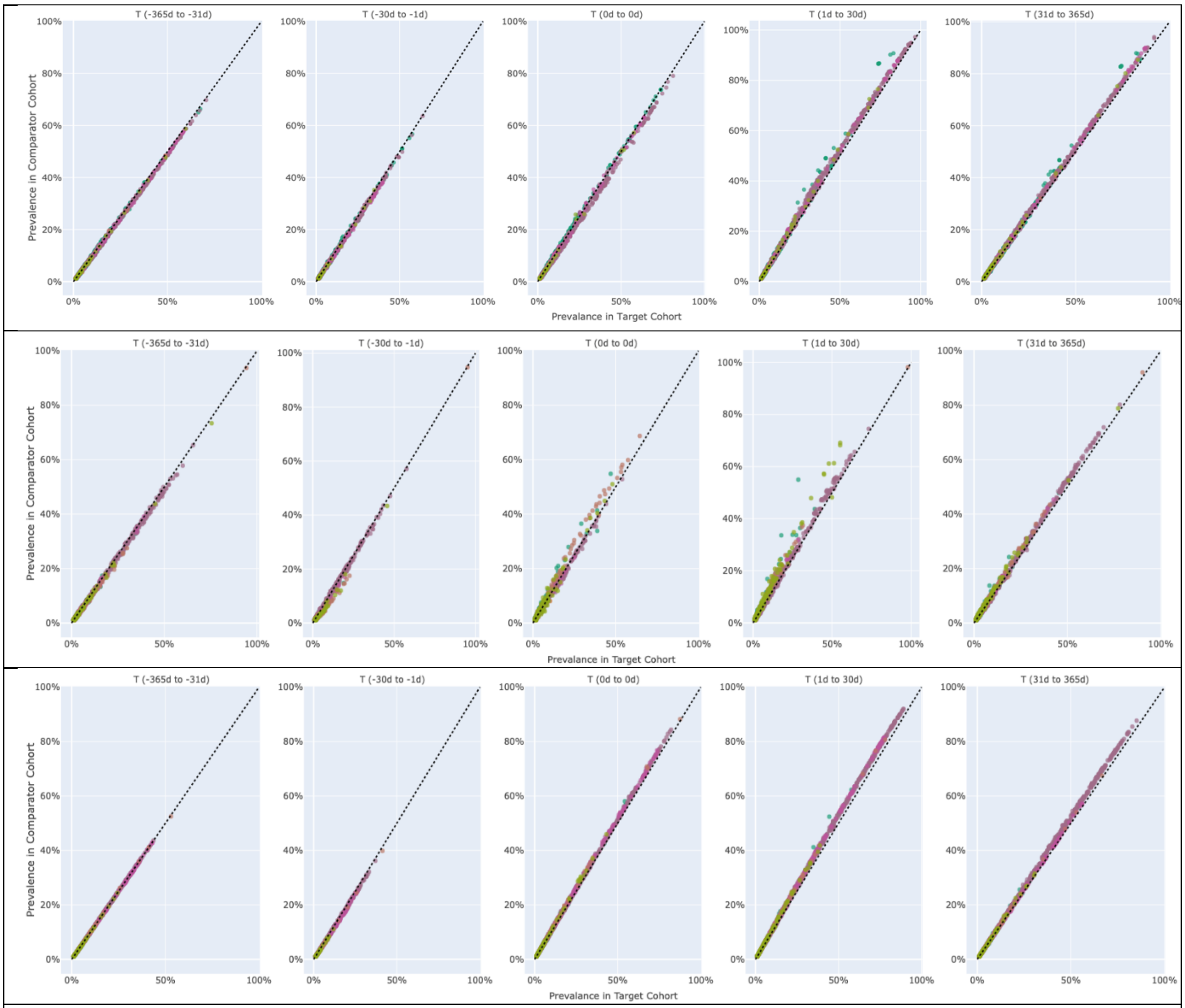

A: Covariate comparison in JDMC of AA (non ruptured) single occurrence (Target cohort, x axis); vs. AA (non ruptured) two occurrences (analysis completed on updated data in April 2024).
B: Covariate comparison in CCAE of AAR single occurrence (Target cohort, x axis) vs. AAR two occurrences (comparator cohort, y axis).
C: Covariate comparison in Optum ® EHR of AD single occurrence (Target cohort, x axis) vs. AD two occurrences (comparator cohort, y axis).

This analysis suggest the same type of medical trajectory is captured in a cohort in using a single condition occurrence code in each of AA (non ruptured), AAR, and AD. In our original analysis, the 'repeated occurrence' AA cohort definition had an error, this analysis was repeated in April 2024 and confirms a similar medical trajectory is represented by a single occurrence definition.

We did note a significant change in cohort sizes when examining the impact of requiring a diagnosis code in the primary position of a record. The relative size of the resulting cohorts when requiring primary position of a code in the claim record is shown below.

**Aortic Disease**	JDMC	Optum ® EHR	IBM CCAE
Aortic Aneurysm (non-ruptured)	29%	19%	6%
Aortic rupture	66%	69%	29%
Aortic dissection	80%	58%	34%

Further, covariate comparison revealed in that in particular with AA, requiring a primary position of the code resulted in cases of surgical AA repair becoming more prevalent in the cohort.
Based on the analysis noted above, we were comfortable with target cohort definitions that required a single aortic disease condition occurrence, with no requirement that be in the primary position in a claims record.

Supplementary method 5. Negative control outcomes

Negative control outcomes are outcomes known to have no association with the target (fluoroquinolone) or comparator (trimethoprim or cephalosporin) cohorts, such that we can assume the true relative risk between the two cohorts is 1. Negative control outcomes were selected using a similar process to that outlined by Voss et al.[16] Once potential negative control candidates were selected, manual clinical review was performed to exclude any pairs that may have a causal relationship or were similar to the study outcome. The top 50 outcomes based on their prevalence were selected. The final list of 50 negative outcomes is described below.

OMOP Concept ID	Outcome Name
4170145	Absence of lung
4093531	Absence of toe
4092879	Absent kidney
42539582	Acquired absence of genital organ
434170	Atypical squamous cells of undetermined significance on cervical Papanicolaou smear
4067069	Callosity
4213540	Cervical somatic dysfunction
443570	Cervicovaginal cytology: Low grade squamous intraepithelial lesion
201613	Chronic nonalcoholic liver disease
43021250	Complication associated with orthopedic device
46269889	Complication due to Crohn's disease
42537730	Coronary artery graft present
436233	Delayed milestone
438021	Disorder due to and following fracture of upper limb
192367	Dysplasia of cervix
4062791	Endocrine, nutritional and metabolic disease complicating pregnancy, childbirth and puerperium
200775	Endometrial hyperplasia
374358	Excess skin of eyelid
4059015	Falls
4264617	Foot-drop
4201388	Gastrostomy present
4166231	Genetic predisposition
4295287	Hypercoagulability state
196473	Hypertrophy of uterus
443447	Iatrogenic hypotension
4344500	Impingement syndrome of shoulder region
441417	Incoordination
4168222	Intra-abdominal and pelvic swelling, mass and lump
196168	Irregular periods
439795	Minimal cognitive impairment
40480893	Nonspecific tuberculin test reaction
438130	Opioid abuse
4022076	Patient dependence on care provider

4141640	Perimenopausal disorder
437092	Physiological development failure
4012231	Poor stream of urine
46286594	Problem related to lifestyle
443274	Psychostimulant dependence
436246	Reduced libido
43021237	Secondary erectile dysfunction
4052226	Sequelae of injuries of lower limb
4125590	Slurred speech
36713918	Somatic dysfunction of lumbar region
4002818	Spasm of back muscles
4008710	Stenosis due to any device, implant AND/OR graft
4201387	Tracheostomy present
42538119	Transplanted heart valve present
444074	Victim of vehicular AND/OR traffic accident
195603	Vulval and/or perineal noninflammatory disorders
4216670	Worried well

Supplementary method 6. Study diagnostics for cohort study

Analyses using observational data can produce misleading estimates as a result of study design and analytic choices. We implemented several study diagnostics using pre-specified decision thresholds to evaluate the reliability of our analyses. These study diagnositics assist with understanding the risk of bias and generalisability of the estimates generated in each database across the network of databases involved in the study. Only results that passed the pre-specified thresholds set for each diagnostics contributed to the meta-analytic estimates.

6A. Covariate Balance

We implemented propensity score matching to account for confounding between treatment groups. Propensity score models included covariates such as age, race, medications, medical conditions, procedure exposure, medical exposure, and laboratory values. of the number of covariates included was between 10,000 to 100,000. Large-scale [20]regularized regression was used to fit the propensity model patients were matched 1:1 between the target and outcome cohorts..

To determine whether the PS matching was sufficient to balance baseline patient characteristics we calculated the standardised mean difference between treatment cohorts after propensity score matching. Covariate balance diagnostic was achieved if all SMDs of predefined covariates in the table were less than 0.1.

Age group	Age was grouped in 5-year intervals
Gender	
Race	
Ethnicity	
Medical history	Acute respiratory disease, Attention deficit hyperactivity disorder, Chronic liver disease, Chronic obstructive lung disease, Crohn's disease, Dementia, Depressive disorder, Diabetes mellitus, Gastroesophageal reflux disease, Gastrointestinal haemorrhage, Human immunodeficiency virus infection, Hyperlipidaemia,Hypertensive disorder, Lesion of liver, Obesity, Osteoarthritis, Pneumonia, Psoriasis, Renal impairment, Rheumatoid arthritis, Schizophrenia, Substance abuse, Ulcerative colitis, Viral hepatitis C, Visual system disorder, Atrial fibrillation, Cerebrovascular disease, Coronary arteriosclerosis, Heart disease, Heart failure, Ischemic heart disease, Peripheral vascular disease, Pulmonary embolism, Venous thrombosis, Hematologic neoplasm, Malignant lymphoma, Malignant neoplasm of anorectum, Malignant neoplastic disease, Malignant tumour of breast, Malignant tumor of colon, Malignant tumor of lung, Malignant tumor of urinary bladder, Primary malignant neoplasm of prostate
Medication use	Agents acting on the renin-angiotensin system, Antibacterials for systemic use, Antidepressants, Antiepileptics, Antiinflammatory and antirheumatic products, Antineoplastic agents, Antipsoriatics, Antithrombotic agents, Beta blocking agents, Calcium channel blockers, Diuretics, Drugs for acid related disorders, Drugs for obstructive airway diseases, Drugs used in diabetes, Immunosuppressants, Lipid modifying agents, Opioids, Psycholeptics, Psychostimulants, Agents used for ADHD and nootropics
Comorbidity index	Charlson Comorbidity Index, CHA_2DS_2-Vasc score, Diabetes Complications Severity Index

6B. Clinical equipoise

Empirical equipoise is assessed by determining the overlap in preference score distribution between the target and comparator cohorts. The preference score distribution is a transformation of the propensity score. High overlap, and greater equipoise ensures that results will be generalisable back to the original cohort. Good equipoise means that even a large propensity score model could not discriminate between preference for two treatments. This is similar to randomised clinical trials where study participants have the same probability of receiving either intervention in a 1:1 randomised trial regardless of their characteristics. at the clinical equipoise diagnostic was achieved if at least 20% of matched patients had a preference score between 0.3 and 0.7.

6C. Systematic error

To determine systematic error or residual bias (due to study design and analytic choices), we estimated systematic error by employing negative controls. Negative controls were determine as outcomes which were not assumed to be associated with either the target or comparator treatment. We included 50 negative controls in our analysis. For each of these outcomes we implement the analysis as per the primary analysis and we compare the estimated result

of the analysis against the “known truth”, in this case we expect the true realative risk to be 1. Positive controls were not utilised as there are no medicines which are known to increase risk of aortic aneurysm or dissection.

Overall systematic error is calculated as the Expected Absolute Systematic Error (EASE) score. EASE is calculated as the expected abs(log(estimated RR)-log (true RR)) across all negative control estimates. If there is sufficiently low systematic error, then the EASE score should be close to 0 which signifies good calibration of our estimate. If EASE score is low, then calibrated and uncalibrated estimates (hazard ratios, p-values, confidence intervals) will be similar indicating little to no systematic error or unmeasured confounding that is not being accounted fore in the results of the study. The systematic bias diagnostic was achieved if the EASE value was less than 0.25.

Study diagnostics: Overall an analysis was considered to have passed study diagnostics if SMDs of predefined covariates were less than 0.1, equipoise was greater than 0.2 and EASE was less than 0.25.

Supplementary table 1. Results of pre-defined study diagnostics

Data source	FQs vs TMP				FQs vs CPH			
	Max SMD[a]	PS overlap[b]	EASE[c]	Overall[d]	Max SMD[a]	PS overlap[b]	EASE[c]	Overall[d]
CUIMC (US)	**0.08**	**0.91**	**0.15**	**PASS**	**0.05**	**0.58**	**0.14**	**PASS**
IBM CCAE (US)	**0.03**	**0.92**	**0.04**	**PASS**	**0.06**	**0.43**	**0.05**	**PASS**
IBM MDCD (US)	**0.03**	**0.84**	**0.03**	**PASS**	**0.03**	**0.44**	**0.04**	**PASS**
Clinformatcs® (US)	**0.04**	**0.92**	**0.04**	**PASS**	**0.06**	**0.47**	**0.07**	**PASS**
Optum® EHR (US)	**0.05**	**0.89**	**0.06**	**PASS**	**0.08**	**0.51**	**0.04**	**PASS**
PharMetrics (US)	**0.03**	**0.91**	**0.03**	**PASS**	**0.05**	**0.45**	**0.03**	**PASS**
VA (US)	**0.03**	**0.85**	**0.05**	**PASS**	**0.05**	**0.47**	**0.05**	**PASS**
TMUCRD (TW)	0.13	0.50	0.50	FAIL	**0.09**	**0.67**	**0.20**	**PASS**
AUSOM (KR)	0.51	0.63	-	FAIL	0.18	0.97	-	FAIL
NHIS-NSC (KR)	0.21	0.77	-	FAIL	0.06	0.95	0.35	FAIL
YUHS (KR)	0.20	0.41	-	FAIL	0.07	0.92	0.47	FAIL
JMDC (JP)	0.13	0.49	-	FAIL	0.04	0.89	0.27	FAIL
Japan Claims (JP)	0.13	0.38	-	FAIL	**0.03**	**0.58**	**0.13**	**PASS**
LPD Australia (AU)	0.13	0.53	-	FAIL	0.14	0.56	-	FAIL

Green colour indicates the result passing diagnostics whereas red coloyr indicates the results without passing diagnostics. The results that passed all three diagnostics are shown in bold.

[a]Balance diagnostics are passed if sufficient covariate balance was achieved if standardized difference of the mean <0.1 for all predefined covariates. [b]Equipoise diagnostics are passed if greater than 20% of patients in both cohorts had PS between 0.3 and 0.7. [c]EASE diagnostics are passed if EASE score is 0.25 or lower. If there are insufficient results for negative control outcomes (fewer than 4), the estimation of EASE becomes unfeasible. [d]Final diagnostics are passed only if all predefined diagnostics are passed.

Abbreviation: FQ, fluoroquinolone; TMP, trimethoprim with or without sulfamethoxazole; CPH, cephalosporin; SMD, standardised difference of mean; PS, preference score; EASE, Expected Absolute Systematic Error; US, United States of America; TW, Taiwan; KR, Republic of Korea; JP, Japan; AU, Australia;

Supplementary table 2. Baseline characteristics of patients in the CUIMC between FQ users and TMP users

Characteristic	Before propensity score matching			After propensity score matching		
	FQ (n=10,157)	TMP (n=5,682)	Std. diff	FQ (n=3,996)	TMP (n=3,996)	Std. diff
Age group, %[a]						
35 - 39	6.1	7.7	-0.06	7.4	8.5	-0.04
40 - 44	6.3	6.7	-0.02	6.9	7.0	0.00
45 - 49	6.7	7.1	-0.02	6.1	7.5	-0.06
50 - 54	7.6	7.5	0.00	7.2	7.7	-0.02
55 - 59	8.9	8.8	0.00	8.0	8.6	-0.02
60 - 64	10.5	9.9	0.02	10.3	9.5	0.03
65 - 69	12.1	12.1	0.00	12.4	11.5	0.03
70 - 74	11.5	11.6	0.00	11.3	11.4	0.00
75 - 79	10.3	10.7	-0.01	10.0	10.7	-0.02
80 - 84	8.6	7.7	0.03	8.5	7.7	0.03
85 - 89	6.6	6.2	0.01	7.3	6.3	0.04
Sex: women, %	67.9	72.0	-0.09	72.5	72.8	0.00
Race, %[b]						
Asian	1.7	1.7	0.00	2.1	1.8	0.03
Black or African American	9.9	10.9	-0.03	9.6	10.8	-0.04
Other Race	0.2	0.1	0.01	0.2	0.1	0.01
White	44.8	45.6	-0.02	46.2	44.0	0.04
Unknown	0.5	0.4	0.01	0.3	0.4	-0.02
Native Hawaiian or Other Pacific Islander	0.3	0.2	0.02	0.4	0.2	0.03
American Indian or Alaska Native	0.1	0.1	0.01	0.1	-0.1	0.03
Ethnicity, %[c]						
Hispanic or Latino	29.0	29.6	-0.01	28.6	30.4	-0.04
Not Hispanic or Latino	45.3	47.0	-0.04	46.9	45.2	0.03
Medical history, %[d]						

Hypertensive disorder	48.9	47.4	0.03	47.2	45.2	0.04
Atrial fibrillation	10.5	11.5	-0.03	11.5	10.5	0.03
Heart failure	10.3	11.3	-0.04	11.4	10.5	0.03
Ischemic heart disease	8.2	9.1	-0.03	8.7	8.6	0.01
Peripheral vascular disease	5.4	5.6	-0.01	5.6	5.0	0.03
Heart valve disorder	9.3	10.7	-0.05	10.3	9.9	0.01
Cerebrovascular disease	9.5	9.8	-0.01	10.1	9.2	0.03
Diabetes mellitus	23.0	23.1	0.00	22.6	21.9	0.02
Hyperlipidemia	35.0	34.1	0.02	34.3	32.8	0.03
Chronic liver disease	4.0	4.4	-0.02	4.4	3.7	0.04
Renal impairment	18.7	20.2	-0.04	19.7	17.6	0.05
Chronic obstructive lung disease	6.3	7.5	-0.05	6.9	6.7	0.01
Crohn's disease	0.6	0.5	0.01	0.6	0.6	0.00
Ulcerative colitis	0.4	0.4	0.01	0.5	0.5	0.01
Dementia	5.4	5.3	0.01	5.6	5.4	0.01
Depressive disorder	13.1	14.4	-0.04	13.9	13.8	0.00
Human immunodeficiency virus infection	1.4	1.9	-0.04	1.7	1.9	-0.01
Psoriasis	0.9	0.7	0.03	1.0	0.7	0.03
Rheumatoid arthritis	1.4	1.6	-0.02	1.7	1.4	0.02
Malignant neoplastic disease	19.5	21.6	-0.05	21.2	19.9	0.03
Medication use, %[e]						
Antithrombotic agents	41.7	44.8	-0.06	44.4	41.7	0.06
Agents acting on the renin-angiotensin system	31.6	30.8	0.02	31.4	30.1	0.03
Beta blocking agents	32.2	33.2	-0.02	32.2	30.5	0.04
Calcium channel blockers	24.9	26.3	-0.03	25.5	24.1	0.03
Diuretics	30.8	32.9	-0.05	31.5	30.5	0.02
Drugs used in diabetes	23.3	26.1	-0.07	24.8	23.9	0.02
Lipid modifying agents	37.2	38.6	-0.03	36.8	36.9	0.00

Antiinflammatory and antirheumatic products	51.4	52.6	-0.02	53.0	51.2	0.04
Immunosuppressants	9.8	12.9	-0.10	10.5	9.6	0.03
Antidepressants	24.5	26.6	-0.05	26.4	25.1	0.03
Charlson comorbidity index[f]	3.5	3.8	-0.05	3.7	3.5	0.05

To account for baseline differences between the two groups, PS-based matching was used. PSs were calculated in each database independently, based on available demographic characteristics, as well as the medical, medication, procedure exposure history, and health service-use behaviours of each database. More detailed balance data before and after PS adjustment can be explored at: https://data.ohdsi.org/FluoroquinoloneAorticAneurysm/

[a]Age groups over 90 were omitted.
[b]The race is reported based on the captured information in the database allowing missing values
[c]The ethnicity is reported based on the captured information in the database allowing missing values
[d]Medical history was identified by coded medical diagnosis within 1 year prior to the cohort entry.
[e]Medication use was identified by medication records within 1 year prior to the cohort entry. Both ATC class-level and ingredient-level drug uses were used to fit the PS model. The only class-level balances of drugs before and after PS matching is reported in this table.
[f]Charlson comorbidity (Romano adaptation) was calculated based on the medical history prior to the cohort entry

Abbreviation: FQ, fluoroquinolone; TMP, trimethoprim with or without sulfamethoxazole; Std.diff, standardized difference; PS, propensity score; CUIMC, Columbia University Irving Medical Center data warehouse

Supplementary table. Baseline characteristics of patients in Clinformatics® between FQ users and TMP users

Characteristic	Before propensity score matching			After propensity score matching		
	FQ (n=953,637)	TMP (n=510,619)	Std. diff	FQ (n=410,842)	TMP (n=410,842)	Std. diff
Age group, %[a]						
35 - 39	5.8	6.5	-0.03	6.5	7.1	-0.03
40 - 44	6.5	6.8	-0.01	6.9	7.3	-0.01
45 - 49	7.2	7.3	0.00	7.3	7.7	-0.01
50 - 54	8.2	7.9	0.01	7.9	8.2	-0.01
55 - 59	8.8	8.5	0.01	8.6	8.8	-0.01
60 - 64	8.7	8.5	0.01	8.5	8.6	0.00
65 - 69	11.5	11.2	0.01	11.1	11.4	-0.01
70 - 74	12.9	13.0	0.00	12.9	12.7	0.01
75 - 79	12.2	12.0	0.00	12.1	11.5	0.02
80 - 84	10.2	10.4	0.00	10.2	9.6	0.02
85 - 89	7.2	7.3	0.00	7.3	6.7	0.02
Sex: women, %	75.8	80.4	-0.11	80.7	81.1	-0.01
Medical history, %[b]						
Hypertensive disorder	59.9	58.8	0.02	58.3	57.2	0.02
Atrial fibrillation	8.8	9.1	-0.01	8.8	8.5	0.01
Heart failure	10.2	10.1	0.00	9.9	9.3	0.02
Ischemic heart disease	9.0	8.6	0.01	8.5	8.0	0.02
Peripheral vascular disease	11.2	11.0	0.01	11.0	10.3	0.02
Heart valve disorder	9.9	9.6	0.01	9.5	9.1	0.01
Cerebrovascular disease	9.8	9.5	0.01	9.4	9.0	0.01
Diabetes mellitus	27.5	26.8	0.01	26.6	25.9	0.02
Hyperlipidemia	55.9	54.5	0.03	54.0	53.2	0.02
Chronic liver disease	2.7	2.4	0.02	2.4	2.3	0.01
Renal impairment	16.8	16.1	0.02	15.7	14.7	0.03

Chronic obstructive lung disease	12.2	12.0	0.00	11.8	11.3	0.01
Crohn's disease	0.5	0.5	0.00	0.5	0.5	0.00
Ulcerative colitis	0.6	0.6	0.00	0.5	0.5	0.00
Dementia	7.4	7.6	-0.01	7.6	6.9	0.03
Depressive disorder	19.0	20.6	-0.04	20.3	19.9	0.01
Human immunodeficiency virus infection	0.2	0.2	0.00	0.2	0.2	0.00
Psoriasis	1.3	1.3	0.00	1.3	1.3	0.00
Rheumatoid arthritis	2.9	2.7	0.01	2.7	2.6	0.01
Malignant neoplastic disease	14.4	14.2	0.01	14.2	13.5	0.02
Medication use, %[c]						
Antithrombotic agents	16.7	16.8	0.00	16.4	15.9	0.01
Agents acting on the renin-angiotensin system	39.7	38.5	0.03	38.2	37.5	0.01
Beta blocking agents	29.8	29.6	0.00	29.3	28.6	0.02
Calcium channel blockers	21.2	20.7	0.01	20.6	20.1	0.01
Diuretics	32.7	32.4	0.01	32.6	31.8	0.02
Drugs used in diabetes	20.2	20.2	0.00	20.1	19.6	0.01
Lipid modifying agents	42.0	41.6	0.01	41.1	40.5	0.01
Antiinflammatory and antirheumatic products	31.5	32.2	-0.02	31.9	31.9	0.00
Immunosuppressants	3.7	3.6	0.00	3.6	3.5	0.01
Antidepressants	31.7	34.2	-0.05	33.9	33.5	0.01
Charlson comorbidity index[d]	3.1	3.1	-0.01	3.0	2.9	0.05

To account for baseline differences between the two groups, PS-based matching was used. PSs were calculated in each database independently, based on available demographic characteristics, as well as the medical, medication, procedure exposure history, and health service-use behaviours of each database. More detailed balance data before and after PS adjustment can be explored at: https://data.ohdsi.org/FluoroquinoloneAorticAneurysm/

[a]Age groups over 90 were omitted; [b]Medical history was identified by coded medical diagnosis within 1 year prior to the cohort entry.
[c]Medication use was identified by medication records within 1 year prior to the cohort entry. Both ATC class-level and ingredient-level drug uses were used to fit the PS model. The only class-level balances of drugs before and after PS matching is reported in this table.
[d]Charlson comorbidity (Romano adaptation) was calculated based on the medical history prior to the cohort entry

Abbreviation: FQ, fluoroquinolone; TMP, trimethoprim with or without sulfamethoxazole; Std.diff, standardized difference; PS, propensity score; Clinformatics ®, Optum's de-identified Clinformatics® Data Mart Database

Supplementary table 4. Baseline characteristics of patients in the IBM CCAE between FQ users and TMP users

Characteristic	Before propensity score matching			After propensity score matching		
	FQ (n=1,117,050)	TMP (n=597,447)	Std. diff	FQ (n=505,839)	TMP (n=505,839)	Std. diff
Age group, %						
35 - 39	12.5	13.8	-0.04	14.2	14.6	-0.01
40 - 44	14.4	15.1	-0.02	15.0	15.3	-0.01
45 - 49	15.9	15.9	0.00	15.9	15.9	0.00
50 - 54	17.9	17.2	0.02	17.3	17.3	0.00
55 - 59	19.0	18.4	0.02	18.3	18.1	0.00
60 - 64	18.6	17.8	0.02	17.8	17.2	0.02
65 - 69	1.6	1.6	0.00	1.6	1.6	0.00
Sex: women, %	82.9	88.7	-0.16	88.7	88.9	-0.01
Medical history, %[a]						
Hypertensive disorder	33.6	32.1	0.03	32.1	31.3	0.02
Atrial fibrillation	1.2	1.2	0.00	1.3	1.2	0.01
Heart failure	1.4	1.4	0.00	1.4	1.3	0.01
Ischemic heart disease	2.2	2.0	0.01	2.0	2.0	0.00
Peripheral vascular disease	1.6	1.5	0.00	1.4	1.4	0.00
Heart valve disorder	3.4	3.1	0.01	3.1	3.0	0.01
Cerebrovascular disease	2.2	2.1	0.01	2.0	1.9	0.01
Diabetes mellitus	14.2	13.4	0.02	13.4	13.0	0.01
Hyperlipidemia	32.8	30.7	0.05	30.2	29.9	0.01
Chronic liver disease	2.1	1.9	0.02	1.8	1.8	0.01
Renal impairment	3.0	2.7	0.02	2.8	2.4	0.02
Chronic obstructive lung disease	2.5	2.4	0.00	2.4	2.3	0.01
Crohn's disease	0.5	0.5	0.00	0.5	0.5	0.00
Ulcerative colitis	0.5	0.5	0.00	0.5	0.4	0.00
Dementia	0.3	0.3	-0.01	0.3	0.3	0.00

Depressive disorder	12.9	14.0	-0.03	13.6	13.6	0.00
Human immunodeficiency virus infection	0.2	0.2	0.00	0.2	0.2	0.00
Psoriasis	1.2	1.1	0.01	1.2	1.1	0.01
Rheumatoid arthritis	1.7	1.5	0.01	1.5	1.4	0.01
Malignant neoplastic disease	7.3	7.0	0.01	7.0	6.7	0.01
Medication use, %[b]						
Antithrombotic agents	6.2	6.0	0.01	5.9	5.6	0.01
Agents acting on the renin-angiotensin system	23.9	22.3	0.04	22.1	21.8	0.01
Beta blocking agents	14.4	13.9	0.01	13.8	13.5	0.01
Calcium channel blockers	9.5	8.8	0.03	8.8	8.6	0.01
Diuretics	20.0	19.8	0.01	19.5	19.5	0.00
Drugs used in diabetes	12.1	11.7	0.01	11.6	11.3	0.01
Lipid modifying agents	23.8	22.3	0.04	22.0	21.8	0.01
Antiinflammatory and antirheumatic products	34.9	35.0	0.00	34.5	34.2	0.01
Immunosuppressants	3.5	3.4	0.00	3.4	3.2	0.01
Antidepressants	29.1	31.2	-0.05	30.7	30.7	0.00
Charlson comorbidity index[c]	1.3	1.3	0.00	1.2	1.2	0.03

To account for baseline differences between the two groups, PS-based matching was used. PSs were calculated in each database independently, based on available demographic characteristics, as well as the medical, medication, procedure exposure history, and health service-use behaviours of each database. More detailed balance data before and after PS adjustment can be explored at: https://data.ohdsi.org/FluoroquinoloneAorticAneurysm/

[a]Medical history was identified by coded medical diagnosis within 1 year prior to the cohort entry.
[b]Medication use was identified by medication records within 1 year prior to the cohort entry. Both ATC class-level and ingredient-level drug uses were used to fit the PS model. The only class-level balances of drugs before and after PS matching is reported in this table.
[c]Charlson comorbidity (Romano adaptation) was calculated based on the medical history prior to the cohort entry

Abbreviation: FQ, fluoroquinolone; TMP, trimethoprim with or without sulfamethoxazole; Std.diff, standardized difference; PS, propensity score; IBM CCAE, IBM Health MarketScan® Commercial Claims and Encounters Database

Supplementary table 5. Baseline characteristics of patients in the IBM MDCD between FQ users and TMP users

Characteristic	Before propensity score matching			After propensity score matching		
	FQ (n=163,429)	TMP (n=112,226)	Std. diff	FQ (n=88,791)	TMP (n=88,791)	Std. diff
Age group, %[a]						
35 - 39	18.7	22.9	-0.10	22.3	23.6	-0.03
40 - 44	14.5	16.5	-0.06	16.3	16.3	0.00
45 - 49	12.7	13.7	-0.03	13.6	13.5	0.00
50 - 54	12.9	12.8	0.00	13.1	12.7	0.01
55 - 59	13.3	12.8	0.02	13.0	12.7	0.01
60 - 64	11.9	10.8	0.03	11.0	10.8	0.01
65 - 69	4.6	3.6	0.05	3.6	3.5	0.00
70 - 74	3.2	2.2	0.07	2.2	2.1	0.01
75 - 79	2.7	1.8	0.06	1.8	1.8	0.00
80 - 84	2.6	1.5	0.08	1.5	1.5	0.00
85 - 89	2.6	1.3	0.09	1.4	1.4	0.00
Sex: women, %	83.3	87.8	-0.13	87.0	87.6	-0.02
Race, %[b]						
Black or African American	27.1	28.2	-0.03	28.1	28.3	0.00
White	59.5	58.3	0.02	58.3	58.1	0.00
Ethnicity, %[c]						
Hispanic or Latino	1.9	2.1	-0.01	2.0	2.1	0.00
Medical history, %[d]						
Hypertensive disorder	61.2	56.0	0.11	56.1	55.4	0.01
Atrial fibrillation	5.2	3.9	0.06	3.8	3.8	0.00
Heart failure	12.2	9.4	0.09	9.3	9.1	0.01
Ischemic heart disease	9.0	7.4	0.06	7.4	7.2	0.01
Peripheral vascular disease	10.1	7.9	0.08	7.8	7.6	0.01
Heart valve disorder	7.7	6.5	0.05	6.4	6.3	0.00

Cerebrovascular disease	9.0	6.9	0.08	6.9	6.8	0.01
Diabetes mellitus	33.4	29.8	0.08	29.6	29.1	0.01
Hyperlipidemia	42.4	38.6	0.08	38.7	38.2	0.01
Chronic liver disease	6.0	5.5	0.02	5.5	5.4	0.00
Renal impairment	14.7	10.9	0.11	10.7	10.3	0.01
Chronic obstructive lung disease	22.3	19.5	0.07	19.4	18.9	0.01
Crohn's disease	0.8	0.7	0.01	0.7	0.6	0.01
Ulcerative colitis	0.5	0.4	0.01	0.4	0.4	0.00
Dementia	6.2	4.1	0.10	4.0	4.0	0.00
Depressive disorder	39.4	39.8	-0.01	39.0	38.7	0.01
Human immunodeficiency virus infection	1.2	1.3	0.00	1.2	1.2	0.00
Psoriasis	1.0	1.0	0.00	1.0	0.9	0.01
Rheumatoid arthritis	3.1	2.7	0.02	2.8	2.7	0.01
Malignant neoplastic disease	8.9	7.4	0.06	7.5	7.2	0.01
Medication use, %[e]						
Antithrombotic agents	23.4	21.0	0.06	20.8	20.1	0.02
Agents acting on the renin-angiotensin system	33.9	32.8	0.02	33.7	32.6	0.03
Beta blocking agents	26.4	25.4	0.02	25.9	24.8	0.03
Calcium channel blockers	18.4	17.8	0.02	17.9	17.5	0.01
Diuretics	33.1	32.5	0.01	32.9	32.1	0.02
Drugs used in diabetes	24.1	23.9	0.00	24.0	23.0	0.02
Lipid modifying agents	32.8	32.1	0.01	32.4	31.5	0.02
Antiinflammatory and antirheumatic products	58.2	61.8	-0.07	61.4	60.6	0.02
Immunosuppressants	3.1	3.0	0.01	2.9	2.8	0.01
Antidepressants	49.9	53.1	-0.06	52.8	51.9	0.02
Charlson comorbidity index[f]	3.4	2.9	0.12	2.9	2.8	0.02

To account for baseline differences between the two groups, PS-based matching was used. PSs were calculated in each database independently, based on available demographic characteristics, as well as the medical, medication, procedure exposure history, and health service-use behaviours of each database. More detailed balance data before and after PS adjustment can be explored at: https://data.ohdsi.org/FluoroquinoloneAorticAneurysm/

[a]Age groups over 90 were omitted.
[b]The race is reported based on the captured information in the database allowing missing values
[c]The ethnicity is reported based on the captured information in the database allowing missing values
[d]Medical history was identified by coded medical diagnosis within 1 year prior to the cohort entry.
[e]Medication use was identified by medication records within 1 year prior to the cohort entry. Both ATC class-level and ingredient-level drug uses were used to fit the PS model. The only class-level balances of drugs before and after PS matching is reported in this table.
[f]Charlson comorbidity (Romano adaptation) was calculated based on the medical history prior to the cohort entry

Abbreviation: FQ, fluoroquinolone; TMP, trimethoprim with or without sulfamethoxazole; Std.diff, standardized difference; PS, propensity score; IBM MDCD, IBM Health MarketScan® Multi-State Medicaid Database

Supplementary table 6. Baseline characteristics of patients in the Optum® EHR between FQ users and TMP users

Characteristic	Before propensity score matching			After propensity score matching		
	FQ	TMP	Std. diff	FQ	TMP	Std. diff
Age group, %[a]						
35 - 39	7.6	9.6	-0.07	9.6	10.4	-0.03
40 - 44	8.0	9.2	-0.04	9.1	9.5	-0.01
45 - 49	8.6	9.5	-0.03	9.6	9.8	-0.01
50 - 54	9.8	10.3	-0.01	10.2	10.5	-0.01
55 - 59	10.6	10.8	-0.01	10.8	11.0	0.00
60 - 64	10.1	10.0	0.00	9.9	10.0	0.00
65 - 69	9.7	9.3	0.02	9.4	9.3	0.00
70 - 74	9.3	8.6	0.02	8.6	8.3	0.01
75 - 79	12.6	10.7	0.06	10.7	10.3	0.01
80 - 84	12.3	10.5	0.06	10.5	9.5	0.03
85 - 89	1.3	1.6	-0.02	1.5	1.4	0.01
Sex: women, %	79.3	84.2	-0.13	84.2	84.8	-0.02
Race, %[b]						
Asian	1.3	1.3	0.00	1.3	1.3	0.00
Black or African American	9.1	9.6	-0.02	9.5	9.7	-0.01
White	84.8	84.4	0.01	84.4	84.1	0.01
Ethnicity, %[c]						
Hispanic or Latino	4.6	4.4	0.01	4.3	4.4	-0.01
Not Hispanic or Latino	89.9	90.5	-0.02	90.4	90.3	0.00
Medical history,%[d]						
Hypertensive disorder	49.4	45.7	0.07	45.2	43.8	0.03
Atrial fibrillation	7.9	6.8	0.04	6.6	6.1	0.02
Heart failure	7.4	6.0	0.06	5.8	5.3	0.03
Ischemic heart disease	6.2	5.3	0.04	5.1	4.7	0.02

Peripheral vascular disease	4.8	4.1	0.03	4.1	3.6	0.03
Heart valve disorder	6.6	5.8	0.04	5.8	5.2	0.02
Cerebrovascular disease	5.3	4.6	0.03	4.5	4.2	0.01
Diabetes mellitus	21.9	19.9	0.05	19.6	18.7	0.02
Hyperlipidemia	42.3	39.7	0.05	39.4	38.1	0.03
Chronic liver disease	2.4	2.1	0.02	2.1	2.0	0.01
Renal impairment	13.2	10.7	0.07	10.2	9.3	0.03
Chronic obstructive lung disease	9.3	8.2	0.04	8.2	7.6	0.02
Crohn's disease	0.6	0.5	0.01	0.5	0.5	0.00
Ulcerative colitis	0.4	0.4	0.01	0.4	0.4	0.01
Dementia	5.0	4.2	0.04	4.1	3.8	0.01
Depressive disorder	18.7	19.5	-0.02	19.2	18.4	0.02
Human immunodeficiency virus infection	0.2	0.2	0.00	0.2	0.2	0.00
Psoriasis	1.0	0.9	0.01	0.9	0.9	0.01
Rheumatoid arthritis	2.1	1.8	0.02	1.8	1.6	0.01
Malignant neoplastic disease	11.2	10.2	0.03	10.1	9.4	0.02
Medication use, %[e]						
Antithrombotic agents	38.7	35.3	0.07	34.8	33.2	0.04
Agents acting on the renin-angiotensin system	35.0	31.8	0.07	31.4	30.4	0.02
Beta blocking agents	31.9	29.3	0.06	28.9	27.6	0.03
Calcium channel blockers	19.2	17.1	0.05	17.0	16.2	0.02
Diuretics	32.2	29.7	0.05	29.3	28.3	0.02
Drugs used in diabetes	21.7	20.0	0.04	19.7	18.7	0.03
Lipid modifying agents	37.4	35.0	0.05	34.4	33.3	0.02
Antiinflammatory and antirheumatic products	55.5	54.3	0.02	53.6	52.4	0.02
Immunosuppressants	3.7	3.7	0.00	3.4	3.2	0.01
Antidepressants	34.3	35.4	-0.02	34.9	34.0	0.02
Charlson comorbidity index[f]	2.1	1.9	0.06	1.9	1.7	0.05

To account for baseline differences between the two groups, PS-based matching was used. PSs were calculated in each database independently, based on available demographic characteristics, as well as the medical, medication, procedure exposure history, and health service-use behaviours of each database. More detailed balance data before and after PS adjustment can be explored at: https://data.ohdsi.org/FluoroquinoloneAorticAneurysm/

[a]Age groups over 90 were omitted.
[b]The race is reported based on the captured information in the database allowing missing values
[c]The ethnicity is reported based on the captured information in the database allowing missing values
[d]Medical history was identified by coded medical diagnosis within 1 year prior to the cohort entry.
[e]Medication use was identified by medication records within 1 year prior to the cohort entry. Both ATC class-level and ingredient-level drug uses were used to fit the PS model. The only class-level balances of drugs before and after PS matching is reported in this table.
[f]Charlson comorbidity (Romano adaptation) was calculated based on the medical history prior to the cohort entry

Abbreviation: FQ, fluoroquinolone; TMP, trimethoprim with or without sulfamethoxazole; Std.diff, standardized difference; PS, propensity score, Optum EHR®, Optum© de-identified Electronic Health Record Dataset

Supplementary table 7. Baseline characteristics of patients in the PharMetrics between FQ users and TMP users

Characteristic	Before propensity score matching			After propensity score matching		
	FQ (n=686,312)	TMP (n=358,621)	Std. diff	FQ (n=358,621)	TMP (n=358,621)	Std. diff
Age group, %						
35 - 39	10.1	11.7	-0.05	11.6	12.0	-0.01
40 - 44	11.0	12.0	-0.03	11.8	12.1	-0.01
45 - 49	13.0	13.5	-0.01	13.5	13.7	0.00
50 - 54	14.9	14.5	0.01	14.7	14.9	-0.01
55 - 59	16.5	16.1	0.01	16.2	16.1	0.00
60 - 64	15.9	15.4	0.01	15.4	15.3	0.00
65 - 69	6.4	5.9	0.02	5.9	5.7	0.00
70 - 74	3.7	3.4	0.02	3.3	3.2	0.01
75 - 79	6.1	5.1	0.04	5.1	4.9	0.01
80 - 84	2.3	2.5	-0.01	2.4	2.2	0.01
Sex: women, %	81.6	86.6	-0.14	86.6	86.8	0.00
Medical history, %[a]						
Hypertensive disorder	40.6	37.1	0.07	37.1	36.4	0.02
Atrial fibrillation	3.3	3.1	0.01	3.0	2.9	0.01
Heart failure	3.0	2.6	0.02	2.6	2.5	0.00
Ischemic heart disease	3.5	3.1	0.02	3.1	3.0	0.01
Peripheral vascular disease	3.1	2.8	0.02	2.7	2.6	0.01
Heart valve disorder	3.3	3.0	0.02	2.9	2.8	0.00
Cerebrovascular disease	2.5	2.3	0.02	2.2	2.1	0.00
Diabetes mellitus	16.3	14.9	0.04	14.9	14.5	0.01
Hyperlipidemia	35.0	31.8	0.07	32.0	31.1	0.02
Chronic liver disease	1.4	1.1	0.02	1.1	1.1	0.00
Renal impairment	6.2	5.1	0.05	5.1	4.7	0.02
Chronic obstructive lung disease	4.5	4.3	0.01	4.1	4.0	0.01

Crohn's disease	0.5	0.5	0.01	0.5	0.5	0.00
Ulcerative colitis	0.4	0.4	0.00	0.4	0.4	0.00
Dementia	1.7	1.6	0.01	1.6	1.5	0.01
Depressive disorder	15.3	16.1	-0.02	16.0	15.8	0.00
Human immunodeficiency virus infection	0.2	0.2	0.01	0.2	0.2	0.00
Psoriasis	1.4	1.3	0.01	1.4	1.3	0.01
Rheumatoid arthritis	1.9	1.7	0.01	1.7	1.7	0.00
Malignant neoplastic disease	8.7	8.3	0.02	8.2	7.8	0.01
Medication use, %[b]						
Antithrombotic agents	10.4	9.5	0.03	9.4	8.9	0.02
Agents acting on the renin-angiotensin system	28.0	25.2	0.06	25.3	24.7	0.01
Beta blocking agents	19.0	17.7	0.03	17.7	17.1	0.02
Calcium channel blockers	12.2	11.0	0.04	11.2	10.8	0.01
Diuretics	22.8	21.4	0.03	21.4	21.0	0.01
Drugs used in diabetes	14.6	13.7	0.03	13.6	13.3	0.01
Lipid modifying agents	28.2	25.8	0.05	25.8	25.2	0.01
Antiinflammatory and antirheumatic products	37.3	36.5	0.02	36.4	35.9	0.01
Immunosuppressants	3.9	3.6	0.01	3.6	3.5	0.01
Antidepressants	29.9	31.7	-0.04	31.4	31.1	0.01
Charlson comorbidity index[c]	1.4	1.3	0.03	1.3	1.2	0.03

To account for baseline differences between the two groups, PS-based matching was used. PSs were calculated in each database independently, based on available demographic characteristics, as well as the medical, medication, procedure exposure history, and health service-use behaviours of each database. More detailed balance data before and after PS adjustment can be explored at: https://data.ohdsi.org/FluoroquinoloneAorticAneurysm/

[a]Medical history was identified by coded medical diagnosis within 1 year prior to the cohort entry.
[b]Medication use was identified by medication records within 1 year prior to the cohort entry. Both ATC class-level and ingredient-level drug uses were used to fit the PS model. The only class-level balances of drugs before and after PS matching is reported in this table.
[c]Charlson comorbidity (Romano adaptation) was calculated based on the medical history prior to the cohort entry

Abbreviation: FQ, fluoroquinolone; TMP, trimethoprim with or without sulfamethoxazole; Std.diff, standardized difference; PS, propensity score; PharMetrics, PharMetrics Plus

Supplementary table 8. Baseline characteristics of patients in the VA between FQ users and TMP users

Characteristic	Before propensity score matching			After propensity score matching		
	FQ (n=243,036)	TMP (n=142,691)	Std. diff	FQ (n=108,202)	TMP (n=108,202)	Std. diff
Age group, %[a]						
35 - 39	2.3	3.4	-0.06	3.3	3.4	-0.01
40 - 44	2.7	3.4	-0.04	3.4	3.4	0.00
45 - 49	3.5	4.2	-0.03	4.1	4.3	-0.01
50 - 54	5.5	6.1	-0.03	6.0	6.3	-0.01
55 - 59	8.7	9.0	-0.01	9.1	9.2	0.00
60 - 64	14.8	14.2	0.01	14.4	14.6	0.00
65 - 69	18.1	17.3	0.02	17.2	17.2	0.00
70 - 74	13.2	13.3	0.00	13.1	12.9	0.01
75 - 79	9.5	9.2	0.01	9.3	9.1	0.01
80 - 84	9.2	8.5	0.03	8.6	8.5	0.00
85 - 89	7.7	7.0	0.03	7.1	7.0	0.00
Sex: women, %	13.1	18.4	-0.15	18.0	17.8	0.00
Race, %[b]						
Asian	0.4	0.4	0.01	0.5	0.4	0.01
Black or African American	21.4	22.1	-0.02	22.0	21.9	0.00
White	70.5	69.6	0.02	69.6	69.7	0.00
Unknown	6.3	6.4	0.00	6.4	6.5	-0.01
Native Hawaiian or Other Pacific Islander	0.8	0.7	0.00	0.8	0.7	0.00
American Indian or Alaska Native	0.7	0.7	0.00	0.7	0.7	0.00
Ethnicity, %[c]						
Hispanic or Latino	6.3	5.8	0.02	5.8	5.8	0.00
Not Hispanic or Latino	91.0	91.5	-0.02	91.4	91.3	0.00
Medical history, %[d]						
Hypertensive disorder	68.7	64.8	0.08	64.9	64.7	0.00

Atrial fibrillation	11.2	9.7	0.05	9.5	9.2	0.01
Heart failure	11.7	9.7	0.06	9.4	9.2	0.01
Ischemic heart disease	15.7	13.5	0.06	13.4	13.3	0.00
Peripheral vascular disease	9.1	8.1	0.04	7.9	7.7	0.01
Heart valve disorder	5.3	4.4	0.04	4.4	4.2	0.01
Cerebrovascular disease	9.9	8.6	0.04	8.6	8.3	0.01
Diabetes mellitus	36.3	34.7	0.03	34.4	34.1	0.01
Hyperlipidemia	58.2	56.3	0.04	56.3	56.4	0.00
Chronic liver disease	4.9	4.5	0.02	4.4	4.3	0.00
Renal impairment	21.4	16.9	0.12	16.6	15.8	0.02
Chronic obstructive lung disease	19.2	18.1	0.03	17.8	17.4	0.01
Crohn's disease	0.4	0.4	0.00	0.4	0.4	0.00
Ulcerative colitis	0.5	0.5	0.01	0.5	0.4	0.00
Dementia	6.8	6.7	0.00	6.5	6.1	0.02
Depressive disorder	26.7	28.6	-0.04	28.0	27.4	0.01
Human immunodeficiency virus infection	0.8	0.8	0.00	0.8	0.8	0.00
Psoriasis	1.4	1.3	0.01	1.4	1.3	0.00
Rheumatoid arthritis	1.4	1.2	0.02	1.2	1.1	0.01
Malignant neoplastic disease	21.9	19.8	0.05	19.8	19.0	0.02
Medication use, %[e]						
Antithrombotic agents	46.2	42.0	0.08	41.3	40.2	0.02
Agents acting on the renin-angiotensin system	47.4	42.5	0.10	42.8	42.8	0.00
Beta blocking agents	42.7	38.8	0.08	38.6	38.1	0.01
Calcium channel blockers	29.0	26.2	0.06	26.2	26.0	0.00
Diuretics	40.5	36.8	0.08	36.8	36.6	0.00
Drugs used in diabetes	32.5	30.5	0.04	30.2	29.8	0.01
Lipid modifying agents	56.9	54.3	0.05	54.2	54.1	0.00
Antiinflammatory and antirheumatic products	54.1	53.8	0.01	53.0	52.3	0.01

Immunosuppressants	2.8	2.7	0.01	2.6	2.5	0.01
Antidepressants	39	40.9	-0.04	40.1	39.3	0.02
Charlson comorbidity index[f]	4.2	3.9	0.07	3.9	3.7	0.03

To account for baseline differences between the two groups, PS-based matching was used. PSs were calculated in each database independently, based on available demographic characteristics, as well as the medical, medication, procedure exposure history, and health service-use behaviours of each database. More detailed balance data before and after PS adjustment can be explored at: https://data.ohdsi.org/FluoroquinoloneAorticAneurysm/

[a]Age groups over 90 were omitted.
[b]The race is reported based on the captured information in the database allowing missing values
[c]The ethnicity is reported based on the captured information in the database allowing missing values
[d]Medical history was identified by coded medical diagnosis within 1 year prior to the cohort entry.
[e]Medication use was identified by medication records within 1 year prior to the cohort entry. Both ATC class-level and ingredient-level drug uses were used to fit the PS model. The only class-level balances of drugs before and after PS matching is reported in this table.
[f]Charlson comorbidity (Romano adaptation) was calculated based on the medical history prior to the cohort entry

Abbreviation: FQ, fluoroquinolone; TMP, trimethoprim with or without sulfamethoxazole; Std.diff, standardized difference; PS, propensity score; VA, Department of Veterans Affairs

Supplementary table 9. Baseline characteristics of patients in the CUIMC between FQ users and CPH users

Characteristic	Before propensity score matching			After propensity score matching		
	FQ (n=10,158)	CPH (n=12,079)	Std. diff	FQ (n=4,867)	CPH (n=4,867)	Std. diff
Age group, %[a]						
35 - 39	6.1	10.0	-0.14	7.8	7.3	0.02
40 - 44	6.3	8.0	-0.07	6.8	7.5	-0.03
45 - 49	6.7	7.4	-0.03	7.0	7.0	0.00
50 - 54	7.6	8.1	-0.02	8.2	8.1	0.00
55 - 59	8.9	8.8	0.00	9.5	8.2	0.04
60 - 64	10.5	9.0	0.05	10.0	9.7	0.01
65 - 69	12.1	10.0	0.07	11.2	11.2	0.00
70 - 74	11.5	9.9	0.05	10.8	11.2	-0.01
75 - 79	10.3	8.5	0.06	9.4	9.4	0.00
80 - 84	8.6	8.1	0.02	8.1	8.2	0.00
85 - 89	6.6	6.8	-0.01	6.2	6.8	-0.03
Sex: women, %	67.9	77.1	-0.21	71.0	70.5	0.01
Race, %[b]						
Asian	1.7	1.2	0.05	1.4	1.5	-0.01
Black or African American	9.9	13.2	-0.10	11.6	11.3	0.01
Other Race	0.2	0.2	0.00	0.3	0.1	0.05
White	44.8	32.2	0.26	37.7	38.2	-0.01
Unknown	0.5	0.6	-0.03	0.5	0.5	0.00
Native Hawaiian or Other Pacific Islander	0.3	0.3	0.00	0.3	0.4	-0.01
American Indian or Alaska Native	0.1	0.1	0.00	0.1	0.1	0.00
Ethnicity, %[c]						
Hispanic or Latino	29.0	43.7	-0.31	36.3	36.2	0.00
Not Hispanic or Latino	45.2	31.1	0.30	37.6	37.7	0.00
Medical history, %[d]						

Hypertensive disorder	48.9	50.7	-0.04	51.2	51.4	0.00
Atrial fibrillation	10.5	11.2	-0.02	11.5	10.9	0.02
Heart failure	10.3	12.1	-0.06	12.1	12.5	-0.01
Ischemic heart disease	8.2	9.3	-0.04	9.0	9.0	0.00
Peripheral vascular disease	5.4	5.0	0.02	5.6	5.7	0.00
Heart valve disorder	9.3	9.4	0.00	9.5	10.7	-0.04
Cerebrovascular disease	9.5	10.7	-0.04	10.5	10.9	-0.01
Diabetes mellitus	23.0	25.7	-0.06	25.4	24.7	0.01
Hyperlipidemia	35.0	31.4	0.08	32.7	34.2	-0.03
Chronic liver disease	4.0	3.9	0.00	4.3	4.8	-0.02
Renal impairment	18.7	19.3	-0.01	20.5	21.5	-0.03
Chronic obstructive lung disease	6.3	6.6	-0.01	6.7	7.0	-0.01
Crohn's disease	0.6	0.3	0.04	0.5	0.4	0.02
Ulcerative colitis	0.4	0.3	0.01	0.4	0.4	-0.01
Dementia	5.4	8.4	-0.12	6.6	7.1	-0.02
Depressive disorder	13.1	14.7	-0.05	14.1	14.9	-0.02
Human immunodeficiency virus infection	1.4	1.4	0.00	1.7	1.5	0.02
Psoriasis	0.9	0.7	0.03	1.0	0.7	0.02
Rheumatoid arthritis	1.4	1.4	0.00	1.4	1.7	-0.02
Malignant neoplastic disease	19.5	13.7	0.16	16.7	17.4	-0.02
Medication use, %[e]						
Antithrombotic agents	41.7	44.3	-0.05	45.5	45.6	0.00
Agents acting on the renin-angiotensin system	31.6	31.1	0.01	32.5	33.0	-0.01
Beta blocking agents	32.2	29.9	0.05	32.4	32.4	0.00
Calcium channel blockers	24.9	25.2	-0.01	26.5	26.2	0.01
Diuretics	30.8	30.7	0.00	32.4	33.7	-0.03
Drugs used in diabetes	23.3	24.8	-0.04	25.4	25.7	-0.01
Lipid modifying agents	37.2	34.5	0.06	36.8	37.2	-0.01

Antiinflammatory and antirheumatic products	51.4	59.4	-0.16	55.9	56.9	-0.02
Immunosuppressants	9.8	7.8	0.07	8.9	10.1	-0.04
Antidepressants	24.5	23.1	0.03	24.9	25.1	-0.01
Charlson comorbidity index[f]	3.55	3.665	-0.03	3.8	3.8	-0.01

To account for baseline differences between the two groups, PS-based matching was used. PSs were calculated in each database independently, based on available demographic characteristics, as well as the medical, medication, procedure exposure history, and health service-use behaviours of each database. More detailed balance data before and after PS adjustment can be explored at: https://data.ohdsi.org/FluoroquinoloneAorticAneurysm/

[a]Age groups over 90 were omitted.
[b]The race is reported based on the captured information in the database allowing missing values
[c]The ethnicity is reported based on the captured information in the database allowing missing values
[d]Medical history was identified by coded medical diagnosis within 1 year prior to the cohort entry.
[e]Medication use was identified by medication records within 1 year prior to the cohort entry. Both ATC class-level and ingredient-level drug uses were used to fit the PS model. The only class-level balances of drugs before and after PS matching is reported in this table.
[f]Charlson comorbidity (Romano adaptation) was calculated based on the medical history prior to the cohort entry

Abbreviation: FQ, fluoroquinolone; CPH, cephalosporin; Std.diff, standardized difference; PS, propensity score; CUIMC, Columbia University Irving Medical Center data warehouse

Supplementary table 10. Baseline characteristics of patients in the Clinformatics® between FQ users and CPH users

Characteristic	Before propensity score matching			After propensity score matching		
	FQ (n=953,637)	CPH (n=523,864)	Std. diff	FQ (n=274,869)	CPH (n=274,869)	Std. diff
Age group, %[a]						
35 - 39	5.8	4.6	0.05	4.2	4.8	-0.03
40 - 44	6.6	4.4	0.10	4.4	4.7	-0.02
45 - 49	7.3	4.8	0.10	4.9	5.2	-0.01
50 - 54	8.1	5.6	0.10	5.7	6.0	-0.01
55 - 59	8.8	6.4	0.09	6.7	7.0	-0.01
60 - 64	8.8	7.2	0.06	7.5	7.6	0.00
65 - 69	11.6	10.5	0.04	11.0	11.4	-0.01
70 - 74	12.9	13.9	-0.03	14.1	14.0	0.00
75 - 79	12.1	14.2	-0.06	14.7	14.0	0.02
80 - 84	10.1	14.4	-0.13	14.2	13.4	0.02
85 - 89	7.1	12.7	-0.19	11.4	10.8	0.02
90 - 94	0.7	1.3	-0.06	1.1	1.2	0.00
Sex: women, %	75.7	76.4	-0.01	76.8	76.8	0.00
Medical history, %[b]						
Hypertensive disorder	59.9	70.3	-0.22	69.1	67.8	0.03
Atrial fibrillation	8.7	14.8	-0.19	13.9	13.4	0.01
Heart failure	10.1	16.3	-0.18	15.5	14.8	0.02
Ischemic heart disease	8.9	12.7	-0.12	12.0	11.7	0.01
Peripheral vascular disease	11.1	16.2	-0.15	15.8	14.9	0.03
Heart valve disorder	9.7	13.8	-0.13	13.2	12.8	0.01
Cerebrovascular disease	9.6	13.8	-0.13	13.3	12.7	0.02
Diabetes mellitus	27.3	33.8	-0.14	32.9	32.1	0.02
Hyperlipidemia	55.7	61.4	-0.12	60.6	59.7	0.02
Chronic liver disease	2.6	3.0	-0.02	3.0	2.9	0.01

Renal impairment	16.8	25.7	-0.22	24.5	23.1	0.03
Chronic obstructive lung disease	12.0	16.7	-0.14	16.2	15.6	0.02
Crohn's disease	0.5	0.6	-0.01	0.6	0.6	0.00
Ulcerative colitis	0.6	0.7	-0.01	0.7	0.7	0.00
Dementia	7.1	12.9	-0.19	11.5	11.1	0.01
Depressive disorder	18.9	25.0	-0.15	23.6	23.0	0.01
Human immunodeficiency virus infection	0.2	0.3	-0.01	0.3	0.3	0.00
Psoriasis	1.4	1.4	0.00	1.4	1.4	0.01
Rheumatoid arthritis	2.9	3.8	-0.05	3.7	3.5	0.01
Malignant neoplastic disease	14.4	17.4	-0.08	17.7	16.7	0.03
Medication use, %[c]						
Antithrombotic agents	16.5	24.8	-0.21	23.0	22.5	0.01
Agents acting on the renin-angiotensin system	39.9	43.4	-0.07	44.1	43.0	0.02
Beta blocking agents	29.8	35.5	-0.12	36.0	34.7	0.03
Calcium channel blockers	21.2	25.2	-0.10	25.4	24.6	0.02
Diuretics	32.6	36.7	-0.09	37.5	36.4	0.02
Drugs used in diabetes	20.1	23.9	-0.09	23.8	23.0	0.02
Lipid modifying agents	41.8	45.6	-0.08	46.3	45.0	0.03
Antiinflammatory and antirheumatic products	31.8	33.5	-0.04	32.8	32.3	0.01
Immunosuppressants	3.7	4.3	-0.03	4.4	4.2	0.01
Antidepressants	31.7	36.3	-0.10	36.3	35.3	0.02
Charlson comorbidity index[d]	3.0	4.2	-0.33	4.0	3.8	0.06

To account for baseline differences between the two groups, PS-based matching was used. PSs were calculated in each database independently, based on available demographic characteristics, as well as the medical, medication, procedure exposure history, and health service-use behaviours of each database. More detailed balance data before and after PS adjustment can be explored at: https://data.ohdsi.org/FluoroquinoloneAorticAneurysm/

[a]Age groups over 90 were omitted.
[b]Medical history was identified by coded medical diagnosis within 1 year prior to the cohort entry.
[c]Medication use was identified by medication records within 1 year prior to the cohort entry. Both ATC class-level and ingredient-level drug uses were used to fit the PS model. The only class-level balances of drugs before and after PS matching is reported in this table.
[d]Charlson comorbidity (Romano adaptation) was calculated based on the medical history prior to the cohort entry

Abbreviation: FQ, fluoroquinolone; CPH, cephalosporin; Std.diff, standardized difference; PS, propensity score; Clinformatics®, Optum's de-identified Clinformatics® Data Mart Database

Supplementary table 11. Baseline characteristics of patients in the IBM CCAE between FQ users and CPH users

Characteristic	Before propensity score matching			After propensity score matching		
	FQ (n=1,117,050)	CPH (n=382,884)	Std. diff	FQ (n=222,632)	CPH (n=222,632)	Std. diff
Age group, %						
35 - 39	12.5	14.3	-0.05	12.9	14.1	-0.03
40 - 44	14.5	14.0	0.01	13.4	13.9	-0.01
45 - 49	15.8	14.8	0.03	14.7	14.8	0.00
50 - 54	18.0	16.8	0.03	16.9	17.0	0.00
55 - 59	19.1	18.7	0.01	19.4	18.8	0.01
60 - 64	18.5	19.4	-0.02	20.7	19.6	0.03
65 - 69	1.7	1.9	-0.01	1.9	1.8	0.01
Sex: women, %	83.0	83.4	-0.01	84.7	84.6	0.00
Medical history, %[a]						
Hypertensive disorder	33.7	38.8	-0.10	38.7	37.4	0.03
Atrial fibrillation	1.3	2.0	-0.06	2.1	2.0	0.01
Heart failure	1.4	2.4	-0.07	2.4	2.3	0.01
Ischemic heart disease	2.2	3.0	-0.05	3.0	2.9	0.01
Peripheral vascular disease	1.6	2.2	-0.04	2.2	2.1	0.01
Heart valve disorder	3.3	4.3	-0.05	4.4	4.2	0.01
Cerebrovascular disease	2.2	2.9	-0.04	3.0	2.8	0.01
Diabetes mellitus	14.2	17.9	-0.10	17.8	16.9	0.02
Hyperlipidemia	32.9	34.6	-0.04	34.8	34.0	0.02
Chronic liver disease	2.2	2.7	-0.03	2.8	2.5	0.01
Renal impairment	3.0	5.3	-0.12	5.3	4.8	0.02
Chronic obstructive lung disease	2.5	3.3	-0.05	3.5	3.2	0.01
Crohn's disease	0.5	0.7	-0.02	0.7	0.7	0.00
Ulcerative colitis	0.5	0.6	-0.01	0.6	0.6	0.01
Dementia	0.3	0.4	-0.03	0.4	0.4	0.00

Depressive disorder	12.8	15.8	-0.09	15.7	15.1	0.01
Human immunodeficiency virus infection	0.2	0.4	-0.03	0.3	0.3	-0.01
Psoriasis	1.2	1.4	-0.02	1.4	1.3	0.00
Rheumatoid arthritis	1.7	2.1	-0.03	2.3	2.1	0.02
Malignant neoplastic disease	7.2	8.4	-0.05	9.0	8.3	0.03
Medication use, %[b]						
Antithrombotic agents	6.2	9.5	-0.12	9.3	8.8	0.02
Agents acting on the renin-angiotensin system	24.1	26.0	-0.04	26.0	25.3	0.02
Beta blocking agents	14.4	16.6	-0.06	17.2	16.2	0.03
Calcium channel blockers	9.4	10.9	-0.05	10.9	10.5	0.01
Diuretics	20.1	22.4	-0.06	22.3	21.7	0.01
Drugs used in diabetes	12.1	15.2	-0.09	14.8	14.3	0.02
Lipid modifying agents	23.8	25.0	-0.03	25.3	24.4	0.02
Antiinflammatory and antirheumatic products	34.9	43.3	-0.17	41.6	40.2	0.03
Immunosuppressants	3.6	4.5	-0.05	4.9	4.4	0.02
Antidepressants	29.2	33.0	-0.08	33.3	32.0	0.03
Charlson comorbidity index[c]	1.3	1.7	-0.21	1.7	1.6	0.05

To account for baseline differences between the two groups, PS-based matching was used. PSs were calculated in each database independently, based on available demographic characteristics, as well as the medical, medication, procedure exposure history, and health service-use behaviours of each database. More detailed balance data before and after PS adjustment can be explored at: https://data.ohdsi.org/FluoroquinoloneAorticAneurysm/

[a]Medical history was identified by coded medical diagnosis within 1 year prior to the cohort entry.
[b]Medication use was identified by medication records within 1 year prior to the cohort entry. Both ATC class-level and ingredient-level drug uses were used to fit the PS model. The only class-level balances of drugs before and after PS matching is reported in this table.
[c]Charlson comorbidity (Romano adaptation) was calculated based on the medical history prior to the cohort entry

Abbreviation: FQ, fluoroquinolone; CPH, cephalosporin; Std.diff, standardized difference; PS, propensity score; IBM CCAE, IBM Health MarketScan® Commercial Claims and Encounters Database

Supplementary table 12. Baseline characteristics of patients in the IBM MDCD between FQ users and CPH users

Characteristic	Before propensity score matching			After propensity score matching		
	FQ (n=163,429)	CPH (n=166,369)	Std. diff	FQ (n=67,180)	CPH (n=67,180)	Std. diff
Age group, %[a]						
35 - 39	18.7	16.1	0.07	17.6	18.2	-0.01
40 - 44	14.5	11.5	0.09	12.9	13.1	-0.01
45 - 49	12.7	9.9	0.09	11.3	11.4	0.00
50 - 54	12.9	10.5	0.08	11.9	11.9	0.00
55 - 59	13.3	11.3	0.06	12.7	12.6	0.00
60 - 64	11.9	10.8	0.03	11.7	11.7	0.00
65 - 69	4.6	7.0	-0.10	5.7	5.3	0.02
70 - 74	3.2	5.9	-0.12	4.3	4.2	0.01
75 - 79	2.7	5.4	-0.13	3.7	3.7	0.00
80 - 84	2.6	5.1	-0.13	3.7	3.7	0.00
85 - 89	2.6	5.8	-0.16	3.8	3.7	0.01
Sex: women, %	83.3	82.6	0.02	83.0	82.8	0.01
Race, %[b]						
Black or African American	27.1	29.4	-0.05	26.6	27.6	-0.02
White	59.5	58.9	0.01	60.4	60.3	0.00
Ethnicity, %[c]						
Hispanic or Latino	1.9	1.8	0.01	1.9	1.8	0.01
Medical history, %[d]						
Hypertensive disorder	61.2	67.5	-0.13	63.7	63.5	0.00
Atrial fibrillation	5.2	8.5	-0.13	6.8	6.7	0.00
Heart failure	12.2	17.2	-0.14	15.0	14.7	0.01
Ischemic heart disease	9.0	11.4	-0.08	10.4	10.3	0.00
Peripheral vascular disease	10.1	13.7	-0.11	12.1	11.9	0.00
Heart valve disorder	7.7	9.5	-0.06	8.8	8.7	0.00

Cerebrovascular disease	9.0	12.1	-0.10	10.7	10.5	0.01
Diabetes mellitus	33.4	38.7	-0.11	36.0	35.5	0.01
Hyperlipidemia	42.4	45.7	-0.07	43.4	42.9	0.01
Chronic liver disease	6.0	5.5	0.02	6.0	6.0	0.00
Renal impairment	14.7	22.0	-0.19	18.3	18.1	0.01
Chronic obstructive lung disease	22.3	25.5	-0.07	24.7	23.9	0.02
Crohn's disease	0.8	0.8	0.00	0.9	0.9	0.00
Ulcerative colitis	0.5	0.5	0.00	0.5	0.5	0.00
Dementia	6.2	11.8	-0.20	8.6	8.5	0.00
Depressive disorder	39.4	40.4	-0.02	40.5	40.5	0.00
Human immunodeficiency virus infection	1.2	1.3	-0.01	1.3	1.2	0.01
Psoriasis	1.0	1.0	0.00	1.0	1.0	0.00
Rheumatoid arthritis	3.1	3.4	-0.02	3.2	3.2	0.00
Malignant neoplastic disease	8.9	9.9	-0.03	9.6	9.4	0.01
Medication use, %[e]						
Antithrombotic agents	23.4	26.0	-0.06	25.6	26.0	-0.01
Agents acting on the renin-angiotensin system	33.9	24.2	0.21	30.6	30.5	0.00
Beta blocking agents	26.4	20.3	0.14	25.5	25.2	0.01
Calcium channel blockers	18.4	14.3	0.11	17.7	17.6	0.00
Diuretics	33.1	25.2	0.17	30.9	30.6	0.01
Drugs used in diabetes	24.1	19.1	0.12	23.4	23.0	0.01
Lipid modifying agents	32.8	24.6	0.18	31.0	30.3	0.01
Antiinflammatory and antirheumatic products	58.2	49.7	0.17	54.7	55.2	-0.01
Immunosuppressants	3.1	2.7	0.02	3.2	3.1	0.01
Antidepressants	49.9	38.0	0.24	46.9	46.8	0.00
Charlson comorbidity index[f]	3.4	4.3	-0.26	3.8	3.7	0.03

To account for baseline differences between the two groups, PS-based matching was used. PSs were calculated in each database independently, based on available demographic characteristics, as well as the medical, medication, procedure exposure history, and health service-use behaviours of each database. More detailed balance data before and after PS adjustment can be explored at: https://data.ohdsi.org/FluoroquinoloneAorticAneurysm/

[a]Age groups over 90 were omitted.
[b]The race is reported based on the captured information in the database allowing missing values
[c]The ethnicity is reported based on the captured information in the database allowing missing values
[d]Medical history was identified by coded medical diagnosis within 1 year prior to the cohort entry.
[e]Medication use was identified by medication records within 1 year prior to the cohort entry. Both ATC class-level and ingredient-level drug uses were used to fit the PS model. The only class-level balances of drugs before and after PS matching is reported in this table.
[f]Charlson comorbidity (Romano adaptation) was calculated based on the medical history prior to the cohort entry

Abbreviation: FQ, fluoroquinolone; CPH, cephalosporin; Std.diff, standardized difference; PS, propensity score; IBM MDCD, IBM Health MarketScan® Multi-State Medicaid Database

Supplementary table 13. Baseline characteristics of patients in the Optum® EHR between FQ users and CPH users

Characteristic	Before propensity score matching			After propensity score matching		
	FQ (n=1,093,755)	CPH (n=680,894)	Std. diff	FQ (n=338,470)	CPH (n=338,470)	Std. diff
Age group, %[a]						
35 - 39	7.6	8.5	-0.03	7.4	8.3	-0.03
40 - 44	8.0	7.1	0.03	6.7	7.3	-0.02
45 - 49	8.7	7.1	0.06	7.0	7.4	-0.01
50 - 54	9.7	7.7	0.07	7.9	8.2	-0.01
55 - 59	10.6	8.5	0.07	9.0	9.0	0.00
60 - 64	9.9	8.7	0.04	9.1	9.2	0.00
65 - 69	9.8	9.0	0.03	9.6	9.3	0.01
70 - 74	9.4	9.6	-0.01	10.2	9.6	0.02
75 - 79	12.6	12.6	0.00	13.8	13.1	0.02
80 - 84	12.4	17.3	-0.14	16.9	16.2	0.02
85 - 89	1.3	3.7	-0.16	2.4	2.5	-0.01
Sex: women, %	79.1	79.1	0.00	79.3	79.4	0.00
Race, %[b]						
Asian	1.3	1.2	0.01	1.1	1.2	0.00
Black or African American	9.2	11.9	-0.09	10.2	11.3	-0.04
White	84.7	81.6	0.08	83.7	82.2	0.04
Ethnicity, %[c]						
Hispanic or Latino	4.5	5.5	-0.05	4.8	5.2	-0.02
Not Hispanic or Latino	90.1	89.0	0.04	89.7	89.0	0.03
Medical history, %[d]						
Hypertensive disorder	49.4	56.6	-0.14	55.7	53.6	0.04
Atrial fibrillation	7.8	12.4	-0.15	11.6	11.1	0.02
Heart failure	7.4	12.0	-0.16	11.1	10.5	0.02

Ischemic heart disease	6.3	9.3	-0.11	8.6	8.2	0.01
Peripheral vascular disease	4.7	6.6	-0.08	6.5	5.9	0.03
Heart valve disorder	6.7	9.6	-0.11	9.1	8.5	0.02
Cerebrovascular disease	5.4	7.3	-0.08	6.9	6.6	0.01
Diabetes mellitus	21.8	27.0	-0.12	25.8	24.9	0.02
Hyperlipidemia	42.3	43.6	-0.03	44.2	41.7	0.05
Chronic liver disease	2.4	2.9	-0.03	2.8	2.7	0.01
Renal impairment	13.2	20.3	-0.19	18.6	17.7	0.02
Chronic obstructive lung disease	9.3	12.6	-0.10	12.3	11.5	0.03
Crohn's disease	0.6	0.6	-0.01	0.6	0.6	0.00
Ulcerative colitis	0.4	0.5	-0.01	0.5	0.5	0.00
Dementia	5.0	8.8	-0.15	7.3	7.3	0.00
Depressive disorder	18.7	21.9	-0.08	21.3	20.2	0.03
Human immunodeficiency virus infection	0.2	0.3	-0.02	0.3	0.3	0.00
Psoriasis	1.0	1.0	0.00	1.0	1.0	0.01
Rheumatoid arthritis	2.1	2.5	-0.03	2.5	2.3	0.01
Malignant neoplastic disease	11.4	12.1	-0.02	12.8	11.8	0.03
Medication use, %[e]						
Antithrombotic agents	38.9	48.7	-0.20	47.3	45.6	0.03
Agents acting on the renin-angiotensin system	35.0	37.0	-0.04	37.8	35.9	0.04
Beta blocking agents	31.9	37.6	-0.12	37.8	35.6	0.05
Calcium channel blockers	19.2	22.8	-0.09	22.7	21.7	0.02
Diuretics	32.2	35.3	-0.06	36.1	34.1	0.04
Drugs used in diabetes	21.7	26.4	-0.11	25.5	24.5	0.02
Lipid modifying agents	37.5	39.3	-0.04	40.2	38.2	0.04
Antiinflammatory and antirheumatic products	55.3	61.4	-0.12	60.3	58.6	0.04
Immunosuppressants	3.7	4.1	-0.02	4.4	4.0	0.02
Antidepressants	34.2	36.4	-0.05	37.1	35.0	0.04

Charlson comorbidity index[f]	2.1	2.8	-0.25	2.7	2.5	0.05

To account for baseline differences between the two groups, PS-based matching was used. PSs were calculated in each database independently, based on available demographic characteristics, as well as the medical, medication, procedure exposure history, and health service-use behaviours of each database. More detailed balance data before and after PS adjustment can be explored at: https://data.ohdsi.org/FluoroquinoloneAorticAneurysm/

[a]Age groups over 90 were omitted.
[b]The race is reported based on the captured information in the database allowing missing values
[c]The ethnicity is reported based on the captured information in the database allowing missing values
[d]Medical history was identified by coded medical diagnosis within 1 year prior to the cohort entry.
[e]Medication use was identified by medication records within 1 year prior to the cohort entry. Both ATC class-level and ingredient-level drug uses were used to fit the PS model. The only class-level balances of drugs before and after PS matching is reported in this table.
[f]Charlson comorbidity (Romano adaptation) was calculated based on the medical history prior to the cohort entry

Abbreviation: FQ, fluoroquinolone; CPH, cephalosporin; Std.diff, standardized difference; PS, propensity score, Optum EHR®, Optum© de-identified Electronic Health Record Dataset

Supplementary table 14. Baseline characteristics of patients in the PharMetrics between FQ users and CPH users

Characteristic	Before propensity score matching			After propensity score matching		
	FQ (n=686,312)	CPH (n=434,870)	Std. diff	FQ (n=211,877)	CPH (n=211,877)	Std. diff
Age group, %						
35 - 39	10.1	11.2	-0.04	10.2	10.7	-0.02
40 - 44	10.9	10.6	0.01	10.1	10.5	-0.01
45 - 49	13.0	11.6	0.04	11.4	11.9	-0.02
50 - 54	14.8	12.9	0.06	13.1	13.4	-0.01
55 - 59	16.6	14.7	0.06	15.2	15.2	0.00
60 - 64	16.1	14.7	0.04	15.8	15.3	0.01
65 - 69	6.3	6.4	0.00	6.8	6.6	0.01
70 - 74	3.7	4.4	-0.04	4.6	4.3	0.01
75 - 79	6.2	8.0	-0.07	8.6	8.0	0.02
80 - 84	2.3	5.6	-0.17	4.3	4.1	0.01
Sex: women, %	81.4	80.4	0.02	82.3	82.1	0.01
Medical history, %[a]						
Hypertensive disorder	40.7	48.2	-0.15	46.4	45.2	0.02
Atrial fibrillation	3.3	6.0	-0.13	5.3	5.1	0.01
Heart failure	3.1	5.5	-0.12	4.9	4.6	0.01
Ischemic heart disease	3.6	5.3	-0.08	4.9	4.6	0.02
Peripheral vascular disease	3.1	4.9	-0.09	4.5	4.2	0.01
Heart valve disorder	3.3	4.6	-0.07	4.3	4.2	0.01
Cerebrovascular disease	2.5	3.7	-0.07	3.5	3.3	0.01
Diabetes mellitus	16.5	21.0	-0.12	19.4	19.0	0.01
Hyperlipidemia	35.0	37.9	-0.06	37.6	36.5	0.02
Chronic liver disease	1.4	1.6	-0.02	1.5	1.6	0.00
Renal impairment	6.2	10.6	-0.16	9.4	8.9	0.02
Chronic obstructive lung disease	4.5	6.4	-0.09	6.1	5.8	0.01

Crohn's disease	0.5	0.6	-0.02	0.6	0.6	0.00
Ulcerative colitis	0.5	0.5	-0.01	0.5	0.5	0.00
Dementia	1.8	3.7	-0.12	2.9	2.9	0.00
Depressive disorder	15.2	18.5	-0.09	17.6	17.3	0.01
Human immunodeficiency virus infection	0.2	0.4	-0.02	0.3	0.3	0.00
Psoriasis	1.5	1.5	0.00	1.6	1.5	0.01
Rheumatoid arthritis	1.9	2.5	-0.04	2.4	2.3	0.01
Malignant neoplastic disease	8.7	10.5	-0.06	10.8	9.9	0.03
Medication use, %[b]						
Antithrombotic agents	10.3	14.5	-0.13	13.8	13.5	0.01
Agents acting on the renin-angiotensin system	28.2	27.1	0.02	29.2	28.8	0.01
Beta blocking agents	18.9	19.8	-0.02	21.4	20.7	0.02
Calcium channel blockers	12.2	12.8	-0.02	14.0	13.5	0.01
Diuretics	22.8	22.9	0.00	24.5	23.9	0.01
Drugs used in diabetes	14.7	15.6	-0.03	16.1	15.9	0.00
Lipid modifying agents	28.1	26.6	0.03	29.2	28.4	0.02
Antiinflammatory and antirheumatic products	37.1	41.6	-0.09	39.9	39.3	0.01
Immunosuppressants	3.9	4.2	-0.01	4.6	4.3	0.01
Antidepressants	29.8	29.2	0.01	31.4	30.7	0.01
Charlson comorbidity index[c]	1.4	1.9	-0.24	1.8	1.7	0.03

To account for baseline differences between the two groups, PS-based matching was used. PSs were calculated in each database independently, based on available demographic characteristics, as well as the medical, medication, procedure exposure history, and health service-use behaviours of each database. More detailed balance data before and after PS adjustment can be explored at: https://data.ohdsi.org/FluoroquinoloneAorticAneurysm/

[a]Medical history was identified by coded medical diagnosis within 1 year prior to the cohort entry.
[b]Medication use was identified by medication records within 1 year prior to the cohort entry. Both ATC class-level and ingredient-level drug uses were used to fit the PS model. The only class-level balances of drugs before and after PS matching is reported in this table.
[c]Charlson comorbidity (Romano adaptation) was calculated based on the medical history prior to the cohort entry

Abbreviation: FQ, fluoroquinolone; CPH, cephalosporin; Std.diff, standardized difference; PS, propensity score; PharMetrics, PharMetrics Plus

Supplementary table 15. Baseline characteristics of patients in the VA between FQ users and CPH users

Characteristic	Before propensity score matching			After propensity score matching		
	FQ (n=243,036)	CPH (n=123,927)	Std. diff	FQ (n=68,155)	CPH (n=68,155)	Std. diff
Age group, %[a]						
35 - 39	2.3	2.5	-0.01	2.2	2.5	-0.02
40 - 44	2.7	2.2	0.03	2.2	2.2	0.00
45 - 49	3.5	2.8	0.04	2.8	2.9	0.00
50 - 54	5.5	4.2	0.06	4.3	4.4	0.00
55 - 59	8.7	7.0	0.06	7.1	7.4	-0.01
60 - 64	14.8	11.9	0.09	12.4	12.5	0.00
65 - 69	18.1	16.6	0.04	17.2	17.0	0.01
70 - 74	13.2	16.1	-0.08	15.5	15.3	0.01
75 - 79	9.5	10.4	-0.03	10.3	10.2	0.00
80 - 84	9.2	10.0	-0.03	10.2	10.0	0.01
85 - 89	7.7	9.2	-0.05	9.2	9.1	0.00
Sex: women, %	13.1	12.2	0.03	12.5	12.9	-0.01
Race, %[b]						
Asian	0.4	0.5	0.00	0.5	0.5	0.00
Black or African American	21.4	22.3	-0.02	21.5	21.7	0.00
White	70.5	69.6	0.02	70.4	70.0	0.01
Unknown	6.3	6.1	0.01	6.1	6.3	-0.01
Native Hawaiian or Other Pacific Islander	0.8	0.8	0.00	0.8	0.8	0.00
American Indian or Alaska Native	0.7	0.7	0.00	0.7	0.7	0.00
Ethnicity, %[c]						
Hispanic or Latino	6.3	6.0	0.01	6.1	6.2	-0.01
Not Hispanic or Latino	91.0	91.4	-0.02	91.2	91.2	0.00
Medical history, %[d]						
Hypertensive disorder	68.7	70.9	-0.05	71.3	70.5	0.02

Atrial fibrillation	11.2	17.3	-0.17	16.4	16.0	0.01
Heart failure	11.7	18.0	-0.18	17.0	16.5	0.02
Ischemic heart disease	15.7	17.7	-0.05	17.6	17.2	0.01
Peripheral vascular disease	9.1	12.1	-0.10	11.8	11.4	0.01
Heart valve disorder	5.3	7.8	-0.10	7.2	7.0	0.00
Cerebrovascular disease	9.9	12.3	-0.08	12.3	11.8	0.01
Diabetes mellitus	36.3	41.6	-0.11	41.2	40.5	0.01
Hyperlipidemia	58.2	57.9	0.00	58.0	57.8	0.00
Chronic liver disease	4.9	5.8	-0.04	5.7	5.5	0.01
Renal impairment	21.4	31.7	-0.23	29.8	28.8	0.02
Chronic obstructive lung disease	19.2	23.3	-0.10	23.0	22.3	0.02
Crohn's disease	0.4	0.4	0.00	0.4	0.4	0.01
Ulcerative colitis	0.5	0.6	-0.01	0.6	0.6	0.00
Dementia	6.8	11.9	-0.18	10.4	10.3	0.00
Depressive disorder	26.7	29.9	-0.07	29.0	28.8	0.01
Human immunodeficiency virus infection	0.8	0.9	-0.01	0.8	0.8	0.01
Psoriasis	1.4	1.4	0.00	1.5	1.4	0.01
Rheumatoid arthritis	1.4	1.5	-0.01	1.6	1.5	0.00
Malignant neoplastic disease	21.9	24.3	-0.06	24.8	23.5	0.03
Medication use, %[e]						
Antithrombotic agents	46.2	59.9	-0.28	57.7	56.4	0.03
Agents acting on the renin-angiotensin system	47.4	48.9	-0.03	49.5	49.1	0.01
Beta blocking agents	42.7	48.5	-0.12	48.3	47.2	0.02
Calcium channel blockers	29.0	32.5	-0.07	32.3	31.7	0.01
Diuretics	40.5	43.9	-0.07	44.0	43.4	0.01
Drugs used in diabetes	32.5	37.9	-0.11	37.6	37.0	0.01
Lipid modifying agents	56.9	59.8	-0.06	59.4	59.1	0.00
Antiinflammatory and antirheumatic products	54.1	60.7	-0.13	59.5	58.6	0.02

Immunosuppressants	2.8	3.7	-0.05	3.5	3.5	0.00
Antidepressants	39.0	44.4	-0.11	43.1	42.9	0.00
Charlson comorbidity index[f]	4.2	5.4	-0.34	5.2	5.0	0.04

To account for baseline differences between the two groups, PS-based matching was used. PSs were calculated in each database independently, based on available demographic characteristics, as well as the medical, medication, procedure exposure history, and health service-use behaviours of each database. More detailed balance data before and after PS adjustment can be explored at: https://data.ohdsi.org/FluoroquinoloneAorticAneurysm/

[a]Age groups over 90 were omitted.
[b]The race is reported based on the captured information in the database allowing missing values
[c]The ethnicity is reported based on the captured information in the database allowing missing values
[d]Medical history was identified by coded medical diagnosis within 1 year prior to the cohort entry.
[e]Medication use was identified by medication records within 1 year prior to the cohort entry. Both ATC class-level and ingredient-level drug uses were used to fit the PS model. The only class-level balances of drugs before and after PS matching is reported in this table.
[f]Charlson comorbidity (Romano adaptation) was calculated based on the medical history prior to the cohort entry

Abbreviation: FQ, fluoroquinolone; CPH, cephalosporin; Std.diff, standardized difference; PS, propensity score; VA, Department of Veterans Affairs

Supplementary table 16. Baseline characteristics of patients in the TMUCRD between FQ users and CPH users

Characteristic	Before propensity score matching			After propensity score matching		
	FQ (n=14,196)	CPH (n=44,560)	Std. diff	FQ (n=7,912)	CPH (n=7,912)	Std. diff
Age group, %[a]						
35 - 39	6.6	10.1	-0.12	8.1	6.8	0.05
40 - 44	6.8	9.2	-0.09	7.6	6.4	0.05
45 - 49	6.8	9.4	-0.10	7.1	6.9	0.00
50 - 54	8.0	10.7	-0.10	8.6	8.1	0.02
55 - 59	9.5	11.5	-0.06	9.7	9.7	0.00
60 - 64	9.4	10.6	-0.04	9.1	10.1	-0.03
65 - 69	9.5	8.8	0.03	9.2	9.5	-0.01
70 - 74	8.4	7.2	0.04	8.1	8.2	0.00
75 - 79	9.2	6.9	0.08	8.9	9.2	-0.01
80 - 84	9.7	6.4	0.12	8.9	9.6	-0.03
85 - 89	9.1	5.5	0.14	8.5	9.5	-0.04
Sex: women, %	61.0	73.2	-0.26	59.3	61.5	-0.04
Race, %[b]						
Unknown	28.4	29.3	-0.02	27.8	28.1	-0.01
Taiwanese	36.3	34.6	0.04	36.0	37.5	-0.03
Ethnicity, %[c]						
Not Hispanic or Latino	100.0	100.0	0.00	100.0	100.0	0.00
Medical history, %[d]						
Hypertensive disorder	27.3	20.0	0.17	24.6	27.7	-0.07
Atrial fibrillation	3.7	2.1	0.10	3.2	3.6	-0.03
Heart failure	9.4	6.2	0.12	8.5	9.5	-0.03
Ischemic heart disease	10.8	8.6	0.07	9.9	11.2	-0.04
Peripheral vascular disease	2.0	1.3	0.06	1.7	1.9	-0.02
Heart valve disorder	4.6	4.4	0.01	4.6	4.6	0.00

Cerebrovascular disease	10.4	6.7	0.13	9.2	10.5	-0.04
Diabetes mellitus	22.8	16.7	0.15	20.6	23.7	-0.07
Hyperlipidemia	20.3	18.0	0.06	18.8	20.0	-0.03
Chronic liver disease	5.0	5.1	0.00	4.8	5.1	-0.02
Renal impairment	10.8	6.2	0.17	9.6	10.6	-0.03
Chronic obstructive lung disease	5.1	2.8	0.12	4.6	4.7	0.00
Crohn's disease	0.0	0.0	0.00	-0.1	-0.1	0.00
Ulcerative colitis	0.0	0.0	0.00	-0.1	0.0	0.00
Dementia	10.3	5.5	0.18	8.9	9.7	-0.03
Depressive disorder	4.5	3.5	0.05	3.9	4.7	-0.04
Human immunodeficiency virus infection	0.1	0.1	-0.01	-0.1	0.1	-0.03
Psoriasis	0.4	0.2	0.03	0.3	0.3	0.00
Rheumatoid arthritis	0.8	0.7	0.00	0.8	0.8	0.00
Malignant neoplastic disease	9.0	6.3	0.10	8.1	9.2	-0.04
Medication use, %[e]						
Antithrombotic agents	27.7	19.3	0.20	25.2	28.0	-0.06
Agents acting on the renin-angiotensin system	26.6	19.1	0.18	24.1	27.3	-0.07
Beta blocking agents	24.7	18.6	0.15	22.6	24.5	-0.05
Calcium channel blockers	29.4	20.3	0.21	26.4	30.4	-0.09
Diuretics	22.6	13.9	0.23	20.0	22.5	-0.06
Drugs used in diabetes	24.5	16.7	0.19	21.7	24.8	-0.07
Lipid modifying agents	20.4	17.1	0.08	18.5	20.7	-0.06
Antiinflammatory and antirheumatic products	62.7	57.5	0.11	60.2	63.1	-0.06
Immunosuppressants	1.1	0.8	0.02	1.0	1.0	-0.01
Antidepressants	13.6	10.1	0.11	12.0	14.2	-0.06
Charlson comorbidity index[f]	2.6	1.8	0.32	2.4	2.6	-0.06

To account for baseline differences between the two groups, PS-based matching was used. PSs were calculated in each database independently, based on available demographic characteristics, as well as the medical, medication, procedure exposure history, and health service-use behaviours of each database. More detailed balance data before and after PS adjustment can be explored at: https://data.ohdsi.org/FluoroquinoloneAorticAneurysm/

[a]Age groups over 90 were omitted.
[b]The race is reported based on the captured information in the database allowing missing values
[c]The ethnicity is reported based on the captured information in the database allowing missing values
[d]Medical history was identified by coded medical diagnosis within 1 year prior to the cohort entry.
[e]Medication use was identified by medication records within 1 year prior to the cohort entry. Both ATC class-level and ingredient-level drug uses were used to fit the PS model. The only class-level balances of drugs before and after PS matching is reported in this table.
[f]Charlson comorbidity (Romano adaptation) was calculated based on the medical history prior to the cohort entry

Abbreviation: FQ, fluoroquinolone; CPH, cephalosporin; Std.diff, standardized difference; PS, propensity score; TMUCRD, Taipei Medical University Clinical Research Database

Supplementary table 17. Baseline characteristics of patients in the Japan Claims between FQ users and CPH users

Characteristic	Before propensity score matching			After propensity score matching		
	FQ (n=24,791)	CPH (n=12,885)	Std. diff	FQ (n=9,569)	CPH (n=9,569)	Std. diff
Age group, %						
35 - 39	11.8	13.1	-0.04	12.3	12.4	0.00
40 - 44	13.3	13.5	-0.01	13.4	14.0	-0.02
45 - 49	14.8	14.3	0.01	14.5	14.4	0.00
50 - 54	17.6	16.5	0.03	17.4	17.3	0.00
55 - 59	19.8	18.6	0.03	19.7	18.7	0.03
60 - 64	12.4	13.0	-0.02	12.2	12.7	-0.01
65 - 69	6.1	6.3	-0.01	6.0	6.0	0.00
70 - 74	4.0	4.3	-0.01	4.0	4.0	0.00
75 - 79	0.3	0.4	-0.01	0.3	0.4	-0.01
80 - 84	0.0	0.0	0.00	0.0	0.0	-0.01
Sex: women, %	58.7	55.1	0.07	60.6	59.5	0.02
Medical history, %[a]						
Hypertensive disorder	11.5	14.7	-0.10	11.2	11.5	-0.01
Atrial fibrillation	0.4	0.5	-0.01	0.4	0.4	0.00
Heart failure	9.5	12.0	-0.08	9.6	9.1	0.02
Ischemic heart disease	10.1	12.8	-0.08	10.1	10.2	0.00
Peripheral vascular disease	1.6	2.0	-0.03	1.6	1.5	0.00
Heart valve disorder	4.6	7.5	-0.12	4.5	4.6	0.00
Cerebrovascular disease	3.4	4.5	-0.06	3.7	3.6	0.00
Diabetes mellitus	30.2	39.1	-0.19	30.3	30.6	-0.01
Hyperlipidemia	15.7	17.4	-0.04	16.1	15.6	0.01
Chronic liver disease	7.6	9.6	-0.07	7.7	7.3	0.01
Renal impairment	4.0	4.9	-0.04	4.0	4.3	-0.01
Chronic obstructive lung disease	1.8	3.4	-0.10	1.6	1.8	-0.01

Crohn's disease	0.1	0.1	0.00	0.1	0.1	0.00
Ulcerative colitis	0.4	0.4	-0.01	0.4	0.5	-0.01
Dementia	0.5	0.6	-0.01	0.6	0.5	0.01
Depressive disorder	3.8	3.7	0.00	3.7	3.6	0.00
Human immunodeficiency virus infection	1.0	1.4	-0.04	0.9	1.0	-0.02
Psoriasis	0.6	0.6	-0.01	0.7	0.5	0.02
Rheumatoid arthritis	5.2	6.1	-0.04	5.4	5.3	0.00
Malignant neoplastic disease	40.1	43.2	-0.06	39.3	39.5	0.00
Medication use, %[b]						
Antithrombotic agents	8.5	11.7	-0.11	8.0	8.3	-0.01
Agents acting on the renin-angiotensin system	16.3	17.4	-0.03	16.0	16.6	-0.01
Beta blocking agents	6.4	6.9	-0.02	6.4	6.4	0.00
Calcium channel blockers	17.1	19.3	-0.06	17.0	17.3	-0.01
Diuretics	5.6	6.4	-0.03	5.1	5.6	-0.02
Drugs used in diabetes	8.6	10.2	-0.05	8.8	9.0	-0.01
Lipid modifying agents	18.4	18.6	-0.01	18.4	18.2	0.00
Antiinflammatory and antirheumatic products	42.5	49.1	-0.13	42.4	42.3	0.00
Immunosuppressants	3.6	3.6	0.00	3.6	3.8	-0.01
Antidepressants	6.0	5.7	0.01	5.9	5.7	0.00
Charlson comorbidity index[c]	3.5	3.9	-0.11	3.5	3.5	0.00

To account for baseline differences between the two groups, PS-based matching was used. PSs were calculated in each database independently, based on available demographic characteristics, as well as the medical, medication, procedure exposure history, and health service-use behaviours of each database. More detailed balance data before and after PS adjustment can be explored at: https://data.ohdsi.org/FluoroquinoloneAorticAneurysm/

[a]Medical history was identified by coded medical diagnosis within 1 year prior to the cohort entry.
[b]Medication use was identified by medication records within 1 year prior to the cohort entry. Both ATC class-level and ingredient-level drug uses were used to fit the PS model. The only class-level balances of drugs before and after PS matching is reported in this table.
[c]Charlson comorbidity (Romano adaptation) was calculated based on the medical history prior to the cohort entry

Abbreviation: FQ, fluoroquinolone; CPH, cephalosporin; Std.diff, standardized difference; PS, propensity score

Supplementary table 18. Incidence rates of primary endpoints

Data Source	FQ vs TMP						FQ vs CPH					
	FQ			TMP			FQ			CPH		
	Original	Matched	Incidence[a]	Original	Matched	Incidence[a]	Original	Matched	Incidence[a]	Original	Matched	Incidence[a]
CUIMC (US)	10,157	3,996	<7.69	5,682	3,996	7.69	10,158	4,867	6.31	12,079	4,867	8.84
IBM CCAE (US)	1,117,050	505,839	0.58	597,447	505,839	0.53	1,117,050	222,632	1.01	382,884	222,632	0.85
IBM MDCD (US)	163,429	88,791	2.87	112,226	88,791	2.09	163,429	67,180	5.19	166,369	67,180	3.52
Clinformatics® (US)	953,637	410,842	3.75	510,619	410,842	4.53	953,637	274,869	6.29	523,864	274,869	7.03
Optum® EHR (US)	1,093,755	478,507	2.23	620,448	478,507	2.34	1,093,755	338,470	4.10	680,894	338,470	4.50
PharMetrics (US)	686,312	358,621	1.12	417,258	358,621	0.96	686,312	211,877	2.41	434,870	211,877	1.84
VA (US)	243,036	108,202	4.46	142,691	108,202	6.40	243,036	68,155	9.25	123,927	68,155	10.46
TMUCRD (TW)	14,195	2,328	<13.60	3,155	2,328	0	14,196	7,912	<4.08	44,560	7,912	4.05
AUSOM (KR)	885	61	0	109	61	0	893	528	0	973	528	0
NHIS-NSC (KR)	13,360	442	0	688	442	0	13,372	5,073	<6.18	7,781	5,073	<6.17
YUHS (KR)	7,476	251	0	557	251	0	7,500	3,581	<8.90	6,323	3,581	<8.97
JMDC (JP)	24,732	722	0	1,121	722	0	24,791	9,569	<3.26	12,885	9,569	<3.26
Japan Claims (JP)	30,901	1,106	0	1,899	1,106	0	30,919	15,060	<2.06	38,663	15,060	0
LPD Australia (AU)	1,240	947	0	8,575	947	0	1,237	889	0	9,840	889	0

[a]Incidence indicates outcome counts per 1000 person-years in matched patients. Person-years were calculated as follow-up days from index date to the earliest of death, event, or 60 days, with incidence rates as events per total follow-up days.

Abbreviations: FQ, fluoroquinolone; TMP, trimethoprim with or without sulfamethoxazole; CPH, cephalosporin; CUIMC, Columbia University Irving Medical Center data warehouse; Clinformatics, Optum's Clinformatics® Data Mart Database; IBM CCAE, IBM Health MarketScan® Commercial Claims and Encounters Database; IBM MDCD, IBM Health MarketScan® Multi-State Medicaid Database; Optum EHR, Optum© de-identified Electronic Health Record Dataset; PharMetrics, PharMetrics Plus; VA, Department of Veterans Affairs; TMUCRD, Taipei Medical University Clinical Research Database; AUSOM, Ajou University School of Medicine; NHIS-NSC, National Health Insurance Service-National Sample Cohort; YUHS, Yonsei University Health System; JMDC, Japan Medical Data Center; LPD Australia, Longitudinal Patient Database in Australia

Supplementary table 19. Prevalence of risk factors (Marfan’s syndrome, Ehlers-Danlos syndrome, Coarctation of aorta, Turner syndrome and bicuspid aortic valve).

**Data Source**	**Condition**	**FQ**	**TMP**
Clinformatics(US)	Ehlers-Danlos syndrome	<2.0%	0.0%
Clinformatics(US)	Coarctation of aorta	<0.5%	<0.5%
Clinformatics(US)	Marfan's syndrome	<0.1%	<0.1%
Clinformatics(US)	Turner syndrome	<0.1%	<0.1%
CUIMC(US)	Marfan's syndrome	<0.1%	0.0%
CUIMC(US)	Coarctation of aorta	<0.1%	0.0%
CUIMC(US)	Ehlers-Danlos syndrome	<0.1%	<0.1%
IBM CCAE(US)	Ehlers-Danlos syndrome	0.0%	<0.1%
**Data Source**	**Condition**	**FQ**	**CPH**
Optum EHR(US)	Turner syndrome	0.0%	0.1%
VA(US)	Ehlers-Danlos syndrome	0.0%	<0.1%
YUHS(KR)	Turner syndrome	0.0%	<0.5%
YUHS(KR)	Marfan's syndrome	0.0%	<0.1%
YUHS(KR)	Bicuspid aortic valve	0.1%	<0.1%

Abbreviation: FQ, fluoroquinolone; TMP, cephalosporin. Values below 0.1% are excluded from this table.

Supplementary figure 1. Kaplan-Meier plots for the risks of the primary outcome

1A. Fluoroquinolone versus trimethoprim with or without sulfamethoxazole

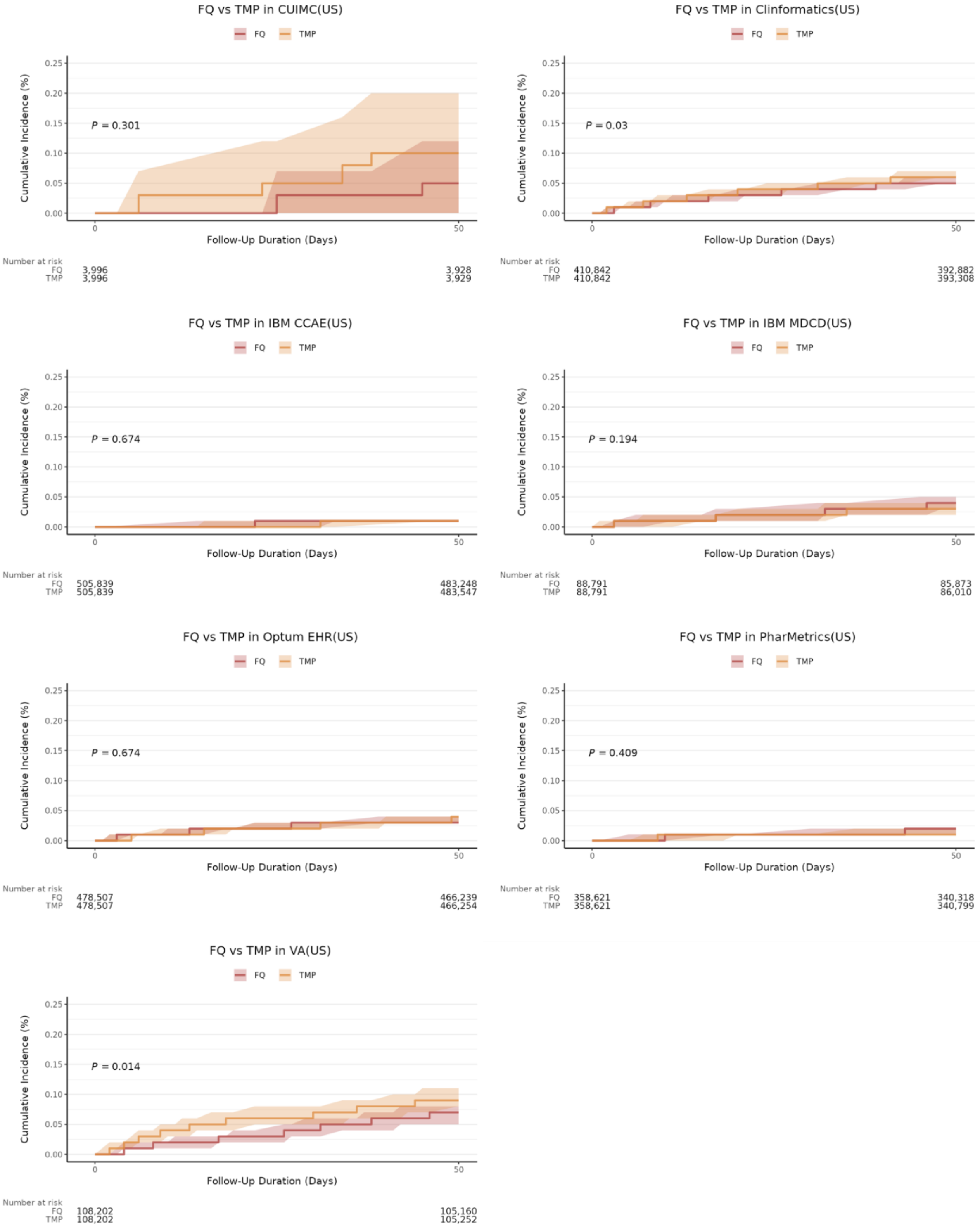

1B. Fluoroquinolone versus cephalosporin

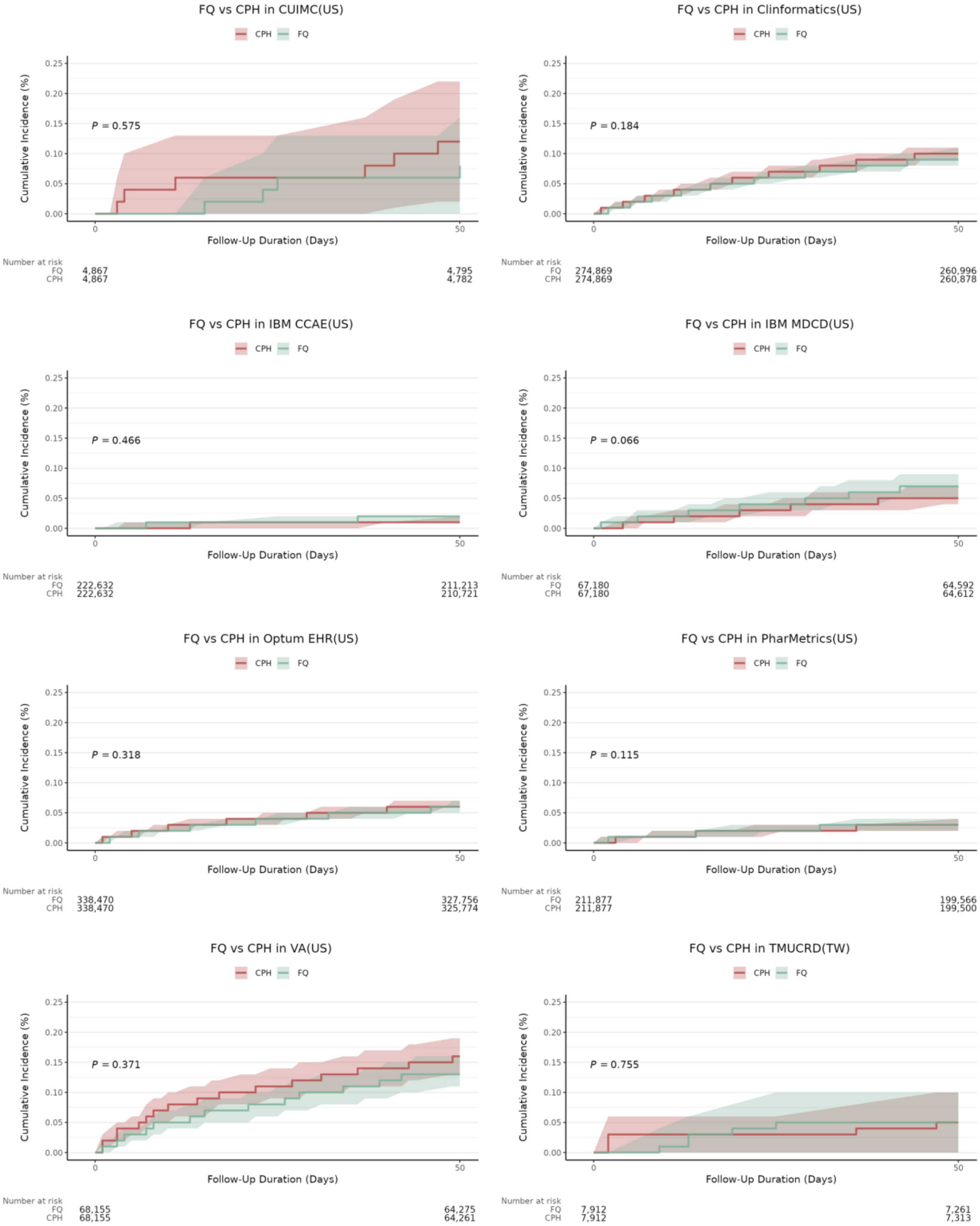

Supplementary figure 2. Systematic error control of effect estimation in the meta-analysis

2A. Systematic error control in comparison of fluoroquinolone vs trimethoprim with or without sulfamethoxazole

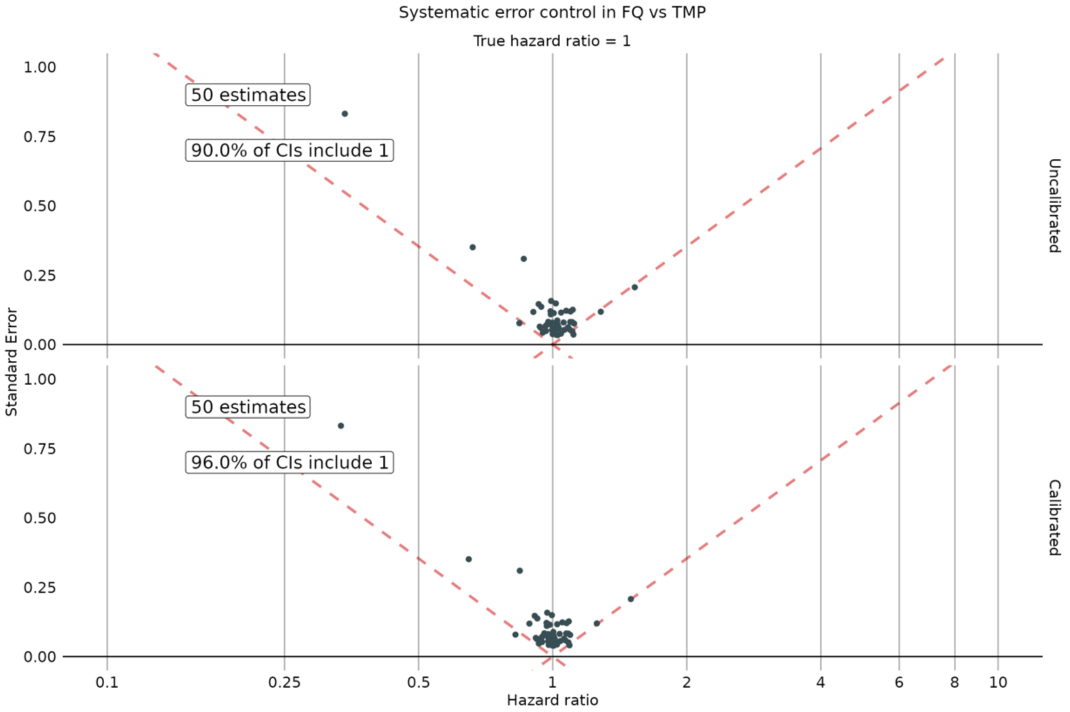

2B. Systematic error control in comparison of fluoroquinolone vs cephalosporin

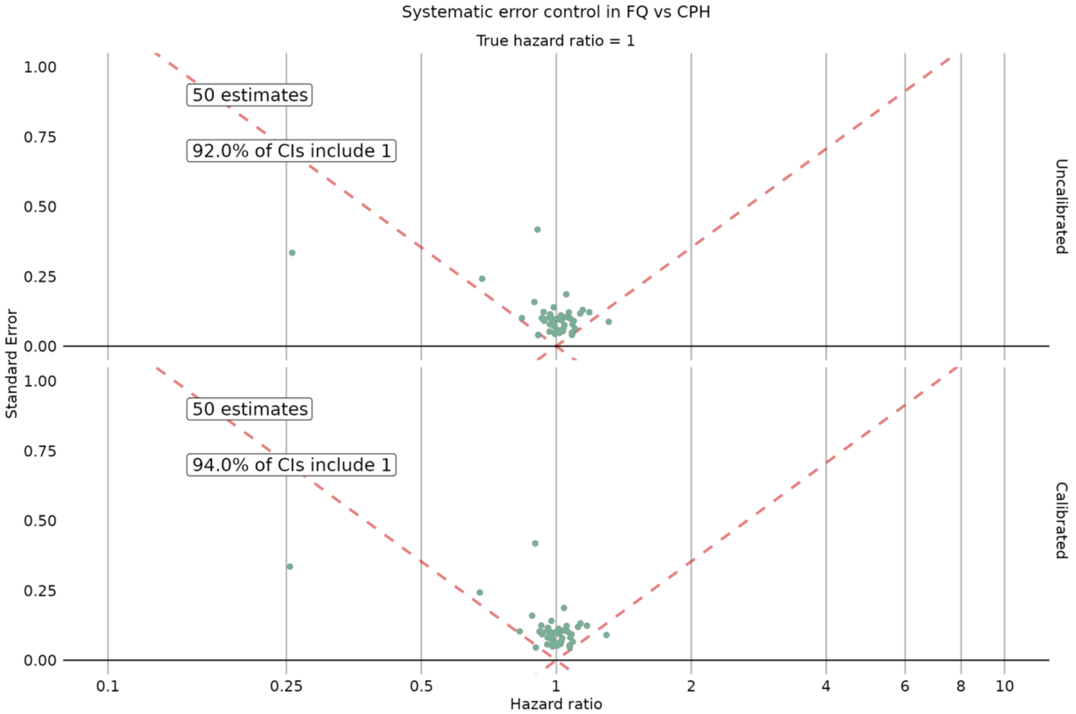

The funnel plots describe the hazard ratio and standard error of each summary estimate of negative control outcomes in the meta-analysis of comparison of fluoroquinolone versus trimethoprim or cephalosporin. In each panel, top plot is the result before calibration and bottom plot is the result after calibration of confidence interval. Overall, nominal 95% confidence intervals cover 91.0% (91/100) and 95.0% (95/100) before and after calibration of confidence interval, respectively.

Supplementary figure 3. The risk of the secondary outcomes in fluoroquinolone vesus trimethoprim with or without sulfamethoxazole or fluoroquinolone versus cephalosprin

3A. Aortic dissection in fluoroquinolone versus trimethoprim with or without sulfamethoxazole

FQ vs TMP

Source	Event Rate FQ	Event Rate TMP	Hazard ratio (95% CI)
Clinformatics(US)	0.41	0.50	0.82(0.48-1.38)
IBM CCAE(US)	0.09	0.07	1.14(0.37-3.49)
IBM MDCD(US)	<0.35	0.49	0.43(0.10-1.74)
Optum EHR(US)	0.24	0.28	0.83(0.44-1.55)
PharMetrics(US)	0.10	0.19	0.54(0.20-1.49)
VA(US)	<0.57	0.79	0.41(0.16-1.10)
**Meta-analysis**	**<0.24**	**0.30**	**0.70(0.47-1.05)**

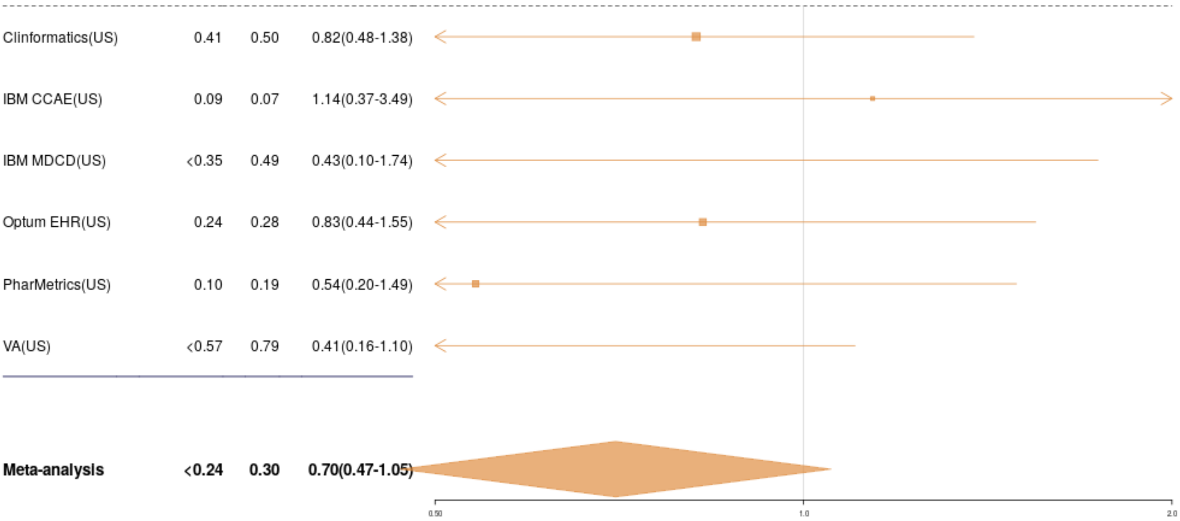

3B. Aortic dissection in fluoroquinolone versus cephalosporin

FQ vs CPH

Source	Event Rate FQ	Event Rate CPH	Hazard ratio (95% CI)
CUIMC(US)	0.00	<6.22	NA
Clinformatics(US)	0.68	0.92	0.74(0.45-1.22)
IBM CCAE(US)	0.17	0.20	0.83(0.27-2.55)
IBM MDCD(US)	<0.46	0.73	0.37(0.09-1.47)
Optum EHR(US)	0.40	0.49	0.80(0.45-1.41)
PharMetrics(US)	0.18	0.44	0.40(0.15-1.04)
VA(US)	<0.91	1.09	0.65(0.26-1.61)
TMUCRD(TW)	<4.07	<4.04	0.32(0.03-4.09)
Japan Claims(JP)	0.00	<2.06	NA
**Meta-analysis**	**<0.43**	**<0.64**	**0.63(0.42-0.94)**

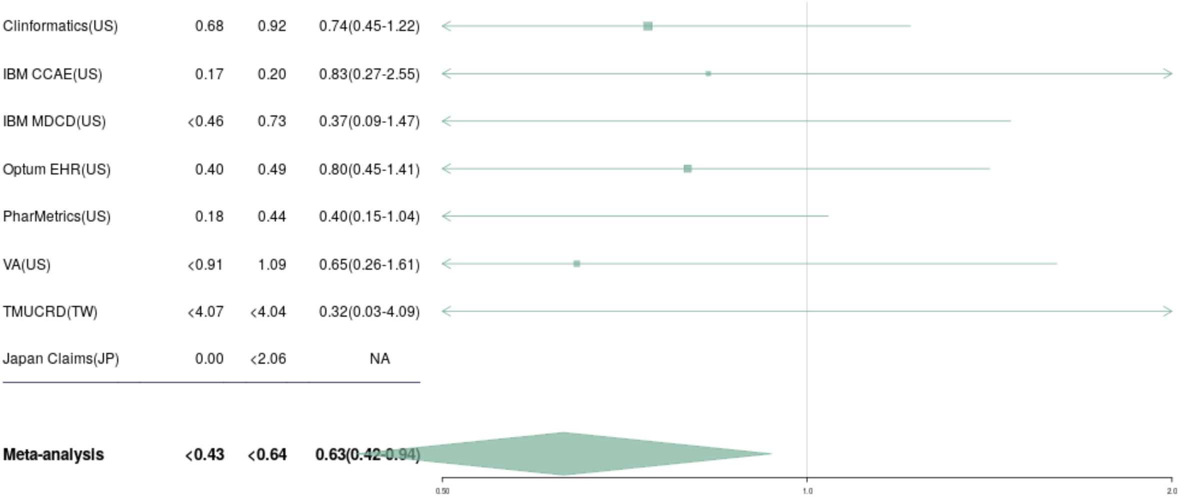

3C. Aortic aneurysm in fluoroquinolone versus trimethoprim with or without sulfamethoxazole

FQ vs TMP

Source	Event Rate FQ	Event Rate TMP	Hazard ratio (95% CI)
CUIMC(US)	<7.68	7.69	0.45(0.08-2.58)
Clinformatics(US)	3.65	4.37	0.83(0.68-1.02)
IBM CCAE(US)	0.54	0.48	1.10(0.71-1.71)
IBM MDCD(US)	2.65	1.88	1.40(0.85-2.30)
Optum EHR(US)	2.08	2.21	0.90(0.71-1.14)
PharMetrics(US)	1.05	0.86	1.21(0.83-1.77)
VA(US)	4.35	6.17	0.68(0.50-0.92)
**Meta-analysis**	**<1.99**	**2.19**	**0.92(0.73-1.17)**

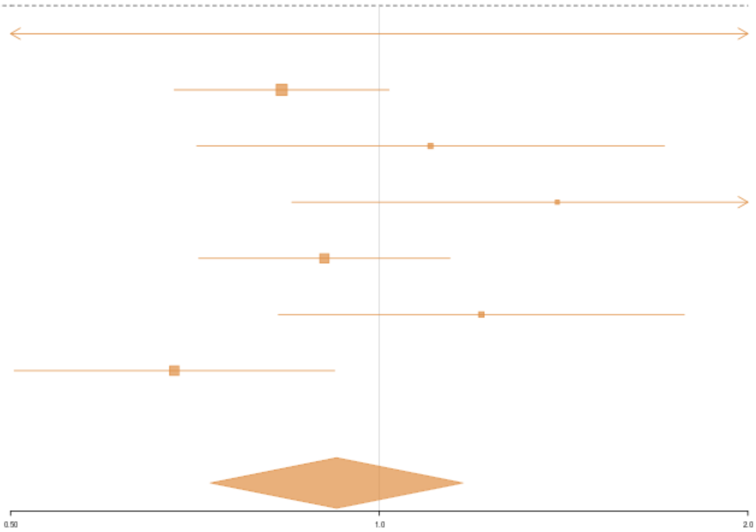

3D. Aortic aneurysm in fluoroquinolone versus cephalosporin

FQ vs CPH

Source	Event Rate FQ	Event Rate CPH	Hazard ratio (95% CI)
CUIMC(US)	7.56	8.83	0.88(0.28-2.73)
Clinformatics(US)	6.15	6.73	0.92(0.74-1.16)
IBM CCAE(US)	0.96	0.73	1.26(0.75-2.14)
IBM MDCD(US)	5.10	3.15	1.60(1.04-2.46)
Optum EHR(US)	3.90	4.32	0.89(0.72-1.09)
PharMetrics(US)	2.35	1.58	1.47(1.03-2.10)
VA(US)	8.97	10.27	0.85(0.64-1.12)
TMUCRD(TW)	<4.08	4.05	0.77(0.19-3.08)
Japan Claims(JP)	<2.06	0.00	NA
**Meta-analysis**	**<3.94**	**3.96**	**1.07(0.83-1.38)**

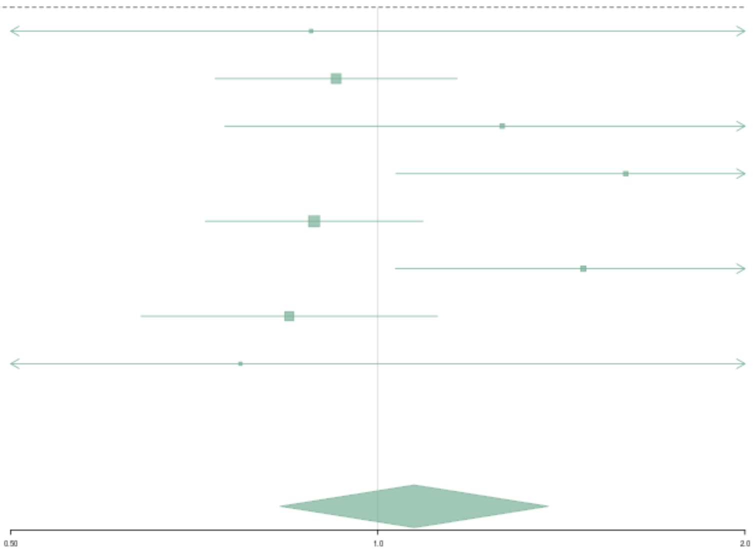

Forest plots depict HR and 95% CI for secondary outcomes. The summary HR were calculated through Bayesian random-effects model. The hazard ratio greater than 1 means increased risk in the fluoroquinolone group compared with trimethoprim or cephalosporin group. The size of data marker indicates the weight of the study. Error bars indicate 95% CIs.

Abbreviations: FQ, fluoroquinolone; TMP, trimethoprim with or without sulfamethoxazole; CPH, cephalosporin; CI, confidence interval; CUIMC, Columbia University Irving Medical Center data warehouse; Clinformatics, Optum's Clinformatics® Data Mart Database; IBM CCAE, IBM Health MarketScan® Commercial Claims and Encounters Database; IBM MDCD, IBM Health MarketScan® Multi-State Medicaid Database; Optum EHR, Optum© de-identified Electronic Health Record Dataset; PharMetrics, PharMetrics Plus; VA, Department of Veterans Affairs; TMUCRD, Taipei Medical University Clinical Research Database; NA, not applicable.

Supplementary figure 4. Hazard ratios (95% CI) across time at risk windows between FQ and TMP or FQ and CPH for each outcome separately and for the composite outcome (AA/AD)

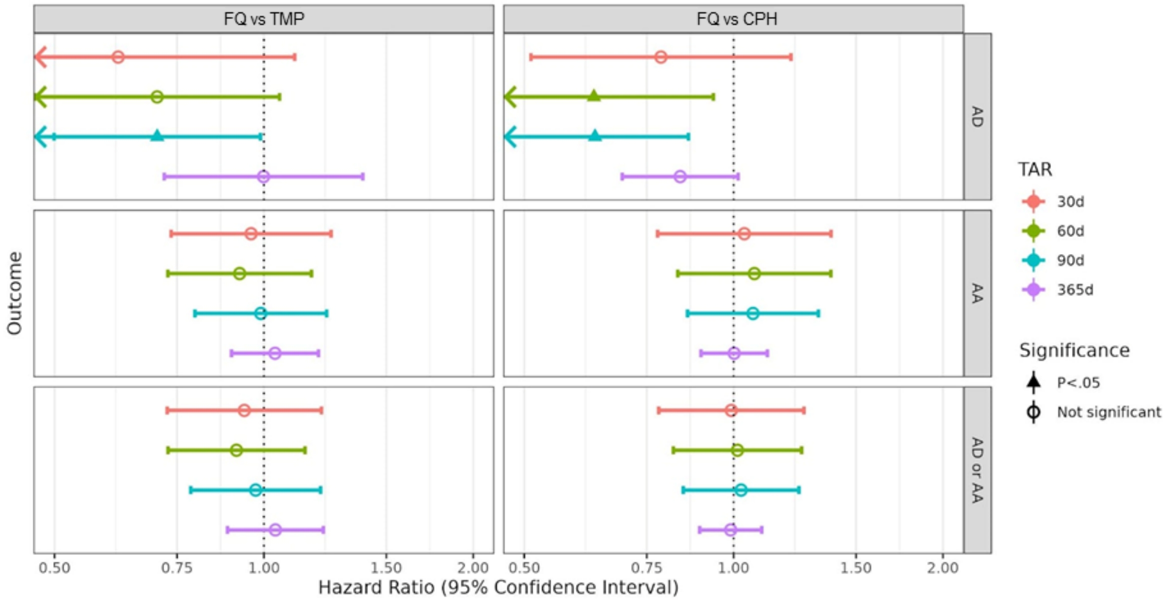

The points indicate HR estimates and the lines their 95% CIs with 30, 60, 90, and 365 times-at-risk. An HR >1 implies a higher risk in the fluoroquinolone group. Open circles represent HRs with CIs that included 1, and triangles represent HRs that were statistically significant.
Abbreviations: CI, confidence interval; FQ, fluoroquinolone; TMP, trimethoprim with or without sulfamethoxazole; CPH, cephalosporin; AD, aortic dissection; AA, aortic aneurysm; TAR, time at risk

Reference

1 Yoon D, Ahn EK, Park MY, *et al.* Conversion and Data Quality Assessment of Electronic Health Record Data at a Korean Tertiary Teaching Hospital to a Common Data Model for Distributed Network Research. *Healthc Inform Res* 2016; **22**: 54–8.

2 You SC, Lee S, Cho S-Y, *et al.* Conversion of National Health Insurance Service-National Sample Cohort (NHIS-NSC) Database into Observational Medical Outcomes Partnership-Common Data Model (OMOP-CDM). *Stud Health Technol Inform* 2017; **245**: 467–70.

3 Lee C-C, Lee MG, Chen Y-S, *et al.* Risk of Aortic Dissection and Aortic Aneurysm in Patients Taking Oral Fluoroquinolone. *JAMA Intern Med* 2015; **175**: 1839–47.

4 Daneman N, Lu H, Redelmeier DA. Fluoroquinolones and collagen associated severe adverse events: a longitudinal cohort study. *BMJ Open* 2015; **5**: e010077.

5 Pasternak B, Inghammar M, Svanström H. Fluoroquinolone use and risk of aortic aneurysm and dissection: nationwide cohort study. *BMJ* 2018; **360**: k678.

6 Lee C-C, Lee MG, Hsieh R, *et al.* Oral Fluoroquinolone and the Risk of Aortic Dissection. *J Am Coll Cardiol* 2018; **72**: 1369–78.

7 Dong Y-H, Chang C-H, Wang J-L, Wu L-C, Lin J-W, Toh S. Association of Infections and Use of Fluoroquinolones With the Risk of Aortic Aneurysm or Aortic Dissection. *JAMA Intern Med* 2020; published online Sept 8. DOI:10.1001/jamainternmed.2020.4192.

8 Gopalakrishnan C, Bykov K, Fischer MA, Connolly JG, Gagne JJ, Fralick M. Association of Fluoroquinolones With the Risk of Aortic Aneurysm or Aortic Dissection. *JAMA Intern Med* 2020; published online Sept 8. DOI:10.1001/jamainternmed.2020.4199.

9 Newton ER, Akerman AW, Strassle PD, Kibbe MR. Association of Fluoroquinolone Use With Short-term Risk of Development of Aortic Aneurysm. *JAMA Surg* 2021; **156**: 264.

10 Brown JP, Wing K, Leyrat C, *et al.* Association Between Fluoroquinolone Use and Hospitalization With Aortic Aneurysm or Aortic Dissection. *JAMA Cardiol* 2023; published online Aug 16. DOI:10.1001/jamacardio.2023.2418.

11 Huh K, Kang M, Jung J. Lack of association between fluoroquinolone and aortic aneurysm or dissection. *Eur Heart J* 2023; : ehad627.

12 Dai X, Yang X, Ma L, Tang G, Pan Y, Hu H. Relationship between fluoroquinolones and the risk of aortic diseases: a meta-analysis of observational studies. *BMC Cardiovasc Disord* 2020; **20**: 49.

13 Son N, Choi E, Chung SY, Han SY, Kim B. Risk of aortic aneurysm and aortic dissection with the use of fluoroquinolones in Korea: a nested case–control study. *BMC Cardiovasc Disord* 2022; **22**: 44.

14 Pasternak B, Inghammar M, Svanström H. Fluoroquinolone use and risk of aortic aneurysm and dissection: nationwide cohort study. *BMJ* 2018; : k678.

15 Schuemie M, Reps J, Black A, *et al.* Health-Analytics Data to Evidence Suite (HADES): Open-Source Software for Observational Research. In: MEDINFO 2023 — The Future Is Accessible. IOS Press, 2024: 966–70.

16 Voss EA, Boyce RD, Ryan PB, van der Lei J, Rijnbeek PR, Schuemie MJ. Accuracy of an automated knowledge base for identifying drug adverse reactions. *J Biomed Inform* 2017; **66**: 72–81.
